# Social determinants of health impact mortality from HCC and cholangiocarcinoma: a population-based cohort study

**DOI:** 10.1097/HC9.0000000000000058

**Published:** 2023-02-09

**Authors:** Lauren D. Nephew, Dipika Gupta, Allie Carter, Archita P. Desai, Marwan Ghabril, Kavish R. Patidar, Eric Orman, Alisha Dziarski, Naga Chalasani

**Affiliations:** 1 Division of Gastroenterology and Hepatology Department of Medicine, Indiana University School of Medicine, Indianapolis, Indiana, USA; 2 Indiana University Simon Comprehensive Cancer Center, Indianapolis, Indiana, USA; 3 Indiana University School of Medicine-Northwest, Gary, Indiana, USA; 4 Department of Biostatistics and Health Data Science, Indiana University School of Medicine, Indianapolis, Indiana, USA; 5 Johns Hopkins University School of Medicine, Baltimore, Maryland, USA; 6 Indiana University School of Medicine, Indiana University Health, Indianapolis, Indiana, USA

## Abstract

The social determinants of health can pose barriers to accessing cancer screening and treatment and have been associated with cancer mortality. However, it is not clear whether area deprivation is independently associated with mortality in HCC and cholangiocarcinoma when controlling for individual-level social determinants of health.

**Approach and Results:**

The cohort included individuals over 18 years old diagnosed with HCC (N=3460) or cholangiocarcinoma (N=781) and reported to the Indiana State Cancer Registry from 2009 to 2017. Area disadvantage was measured using the social deprivation index (SDI). SDI was obtained by linking addresses to the American Community Survey. Individual social determinants of health included race, ethnicity, sex, marital status, and insurance type. The primary outcome was mortality while controlling for SDI and individual social determinants of health by means of Cox proportional hazard modeling. In HCC, living in a neighborhood in the fourth quartile of census-track SDI (most deprived) was associated with higher mortality (HR: 1.14, 95% CI, 1.003–1.30, *p*=0.04) than living in a first quartile SDI neighborhood. Being uninsured (HR: 1.64, 95% CI, 1.30–2.07, *p*<0.0001) and never being married (HR: 1.31, 95% CI, 1.15–1.48, *p*<0.0001) were also associated with mortality in HCC. In cholangiocarcinoma, SDI was not associated with mortality.

**Conclusions:**

Social deprivation was independently associated with mortality in HCC but not cholangiocarcinoma. Further research is needed to better understand how to intervene on both area and individual social determinants of health and develop interventions to address these disparities.

## Graphical abstract

**Figure:**
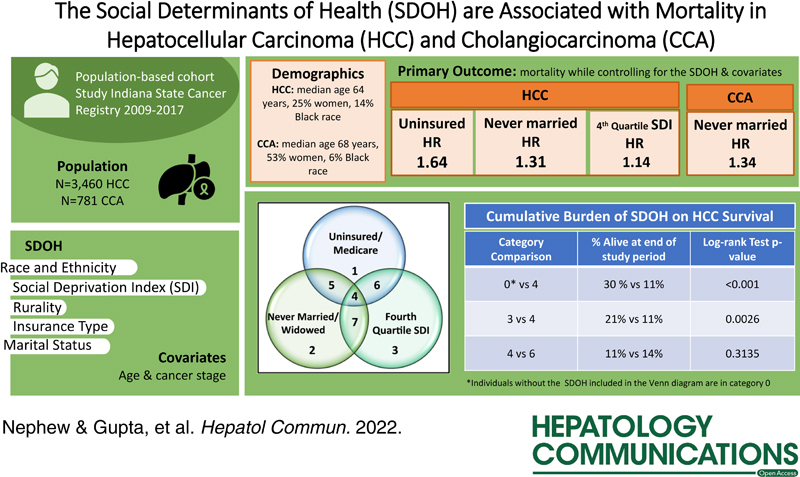

## INTRODUCTION

In 2020 HCC was the third most frequent cause of cancer-related deaths worldwide and is the fastest growing cause of cancer-related death in the US.^1^,^2^ Despite the availability of effective screening and curative treatments, the 5-year survival is only 35% for localized disease.^3^ Although cholangiocarcinoma (CCA) is relatively rare, the incidence in the US is rising. Furthermore, the outcomes for localized disease remain poor, with a 5-year survival of only between 2%–30% and 2%–15% for extrahepatic and intrahepatic disease, respectively.^4^ Despite the overall poor survival rates, both HCC and CCA have the potential to be cured when diagnosed early and curative therapies can be accessed.

The ability to access treatment and navigate cancer care impacts survival and is greatly influenced by the social determinants of health (SDOH).^4^–^6^ The SDOH are the conditions and the places where people live, learn, work, and play and are often grouped into domains that include economic stability, health care quality and access, social and community context, and neighborhood and built environment.^7^,^8^

In 2020, 37.2 million (12.3%) of Americans lived in poverty.^9^ However, this disadvantage is not spread evenly throughout the country’s population. Impoverished individuals are often clustered within specific communities, contributing to an uneven distribution of morbidity and mortality. Composite measures of area disadvantage, like the social deprivation index (SDI), seek to capture multiple components of an area’s socioeconomic status into 1 composite measure. These measures, including SDI, are becoming important tools for targeting cancer control interventions and for adjusting quality measures and payments.^10^,^11^ Indiana is the representative of other states in the country and has 12% of census tracts classified as persistently poor.^12^

Area-level SDOH have begun to be explored in HCC and CCA,^13^–^16^ . Specifically in HCC, in a Surveillance, Epidemiology, and End Results (SEER) database analysis, area-level poverty was associated with mortality (HR: 1.23, 95% CI, 1.16–1.31, *p*<0.001).^13^ In CCA, area-level poverty was associated with poor survival (HR: 0.80, 95% CI, 0.696–0.921).^16^ Notably, these studies measure a single area-level measure of disadvantage, whereas more sensitive, composite measures have not yet been explored. In addition, in HCC the relationship between both area-level and individual SDOH have not been studied. Therefore, in this analysis, we seek to measure the association between SDI and mortality while controlling for individual SDOH in a cohort of patients with HCC and CCA. We hypothesize that SDI is independently associated with mortality when controlling for individual SDOH.

## PATIENTS AND METHODS

### Study population and data source

Adults aged 18 years and older diagnosed with HCC or CCA between January 1, 2009, and December 27, 2017, were included in this study. Demographic, clinical, and individual SDOH data were collected from the Indiana Sate Cancer Registry (ISCR). Indiana mandates reporting of all cases to the cancer registry; therefore, this data set is likely to represent the full burden of HCC and CCA across the state. Furthermore, the ISCR has been designated a US Cancer Statistics Registry for Surveillance by the Centers for Disease Control’s National Program of Cancer Registries. This recognition is based on an annual review of data quality, completeness, and timeliness. All research was conducted in accordance with both the Declarations of Helsinki and Istanbul. All research was approved by the appropriate ethics and/or institutional review committee(s) of Indiana University School of Medicine.

### Exposures

#### Individual SDOH

Exposures of interest included race, ethnicity, sex, insurance type, and marital status. Race, ethnicity, and sex were considered for this analysis as social constructs,^7^ which may be associated with a patient’s ability to access care and thus their overall cancer mortality. Race, sex, and ethnicity are defined by self-report in hospital systems, which report cases to the ISCR. For this analysis, race was classified as Black, White, and other (including but not limited to American Indian, Asian, and unknown race) and ethnicity as Hispanic and non-Hispanic. Sex was classified as men and women. Insurance type is a robust measure of both individual income and health care access.^17^ Insurance was classified as private, Medicare, Medicaid (included Medicare patients with Medicaid supplement), uninsured, and other (ie, Veterans, Indian Public Health Service, and military benefits). Marital status has been associated with cancer outcomes and is hypothesized to be one measure of social support.^18^ Marital status was classified as married (including common law), divorced, separated, single (never married), widowed, and unknown.

#### Area-level SDOH

We investigated county and census-tract level measures of disadvantage, census-tract level poverty, and the impact of rurality on mortality. Because targeting interventions to high-risk counties could be less time intensive than identifying individual census tracts for intervention, we wanted to test the hypothesis that county SDI would be associated with mortality in this cohort. County and neighborhood disadvantage indicators, as well as rural versus urban residence were obtained by linking patients addresses to the 2015 American Community Survey (ACS). The ACS provides data about social, economic, housing, and demographic characteristics for multiple geographic areas.^19^ Census tracts are small areas of ~1200–8000 (average 4000) persons. To measure county and census-tract disadvantage, the SDI was calculated from the ACS. The SDI was developed by Butler et al.^10^ to quantify social inequities across small geographical locations and provides an approach to evaluate their association with health outcomes. The SDI has been used to develop community health interventions and guide adjustments to quality measures and payments. It is a composite measure of 7 demographic characteristics collected at the area level from the ACS: percent living in poverty, percent with <12 years of education, percent single-parent household, percent living in the rented housing unit, percent living in the overcrowded housing unit, percent of households without a car, and percent nonemployed adults under 65 years of age. The SDI was chosen because it is a more sensitive measure than area income alone, but less sensitive than other measures like the area deprivation index, which includes up to 15 ACS variables, offering a balance of sensitivity and specificity. However, we also explored whether neighborhood income below the federal poverty level (FPL) alone was associated with HCC and CCA mortality when controlling for individual SDOH and rurality in this data set. Census-tract income below the FPL was also obtained from the ACS data set.

We used the rural-urban commuting area (RUCA) codes obtained from the ACS to define rural versus urban residence.^20^ All metro census tracts, with RUCA codes 4–9, are considered rural by the Federal Office of Rural Health Policy and is consistent with our previously published work.^20^–^22^ We further classified rural counties into rural-urban adjacent (RUCA codes 4, 6, and 8) and rural not metro adjacent (RUCA codes 5, 7, and 9). Counties with populations of >250,000 were classified as urban (RUCA codes 1, 2, and 3).

### Covariates

Other covariates captured from the ISCR included patient age at the time of diagnosis, date of diagnosis, date of death or last contact, and Surveillance, Epidemiology, and End Results (SEER) cancer stage. SEER stage was classified as localized, regional, distant, and unstaged. Localized stage defines tumors involving a single lobe of the liver. Regional stage defines tumors involving >1 lobe through contiguous growth of a single lesion, extension to adjacent structures, or spread to regional lymph nodes. Distant stage includes tumors that have metastasized, extension of tumor growth to nearby organs, or spread to distant lymph nodes.^23^

### Outcomes

The primary outcome was time to death in HCC and CCA when controlling for census-tract level SDI and individual SDOH. Mortality in this analysis is all-cause mortality. In a secondary analysis, mortality was explored while controlling for: (1) county level SDI in HCC and CCA and (2) census-tract level income below the FPL and individual SDOH in HCC and CCA. A conceptual framework for a potential relationship between the exposures, covariates, and outcomes is provided in Supplemental Figure 1 (http://links.lww.com/HC9/A136) and is adapted from our previous work, as well as Alcaraz et al.^6^ model on addressing social determinants in cancer care.^21^

### Statistical methods

Individual patient characteristics and the SDOH were summarized by mortality status and SDI quartile as means and SDs, medians and quartile ranges, and frequencies and percentages, as appropriate. For the comparison of demographic and clinical characteristics by SDI quartile, continuous variables were tested for differences between the 4 quartiles using ANOVA. Categorical variables were tested for the association between variable and SDI quartile using chi-squared testing.

For the primary and secondary outcome of mortality status controlling for SDI and income below the FPL, univariable and multivariable time-to-event Cox proportional hazard models were performed. Because of the relationship between the exposures, covariates, and mortality proposed in our conceptual framework, all variables were considered for inclusion in the multivariable models if there was no evidence of collinearity. A variance inflation factor of 4 was used as the threshold for suspected collinearity. Because of the suspected collinearity with SDI and multiple other covariates, rurality was not included in the final multivariable models. Ethnicity was not included in the multivariable models for CCA mortality due to sample size (Hispanic subgroup n=3). Interactions were explored between SDI, income below the FPL, and race for all models.

#### Descriptive and subgroup analysis in HCC

Although rurality had the evidence of collinearity with SDI and was not included in multivariable models, we remained interested in exploring the geographic distribution of deaths in Indiana of those individuals in the fourth quartile of SDI given its association with HCC mortality. The relationship between rurality and SDI was explored visually by mapping quartile 4 SDI HCC mortality rates per 100,000 persons.

In HCC, over 70% of the cohort had at least 1 SDOH that was significantly associated with higher mortality. To identify the population most in need of cancer control interventions, subgroup analysis was performed to explore the cumulative burden of 1, 2, or 3 SDOH on HCC mortality. We focused on uninsured/Medicare, never married/widowed, and fourth quartile SDI SDOH as these were significantly associated with mortality in the primary analysis. Only patients with data on all SDOH were included in this analysis. Overall survival was calculated using the Kaplan-Meier method and log-rank testing was performed to compare mortality between patients having zero and 3, 1 and 2, and 2 and 3 SDOH. Because of the statistical concern for multiple comparisons, only 1 comparison was made in each category and the decision was made *a-priori*.

Data summaries were produced using R Statistical Software (version 4.1.0). All analyses were performed using SAS version 9.4 (SAS Institute Inc., Cary, NC). A 0.05 significance level was used for all multivariable analyses.

## RESULTS

### Demographic and clinical characteristics

The study included 3460 patients with HCC and 781 patients with CCA. The median age of the HCC cohort was 64 years [interquartile range (IQR): 57–73] and included 25.2% women and 13.5% individuals of Black race (Table 1). The median age of the CCA cohort was 68 years (IQR: 59–76) and included 52.5% women and 6.4% individuals of Black race (Table 2). Both cohorts had few individuals of Hispanic ethnicity (1.1% of HCC and 0.4% of CCA) (Tables 1, 2). At the time of diagnosis, 45.6% of HCC were localized, compared with 25.2% of CCA.

**TABLE 1:** Demographic, clinical characteristics, neighborhood and individual social determinants of health in patients with HCC by survival status

	Alive (N=576)	Deceased (N=2884)	Total (N=3460)
Age at diagnosis			
Mean (SD)	62.0 (10.2)	65.8 (11.2)	65.2 (11.1)
Median (Q1, Q3)	62.0 (56.8, 68.0)	64.0 (58.0, 74.0)	64.0 (57.0, 73.0)
Range	20.0–90.0	19.0–98.0	19.0–98.0
Sex, n (%)			
Female	160 (27.8)	711 (24.7)	871 (25.2)
Male	416 (72.2)	2173 (75.3)	2589 (74.8)
Race, n (%)			
N-Miss	1	0	1
Black	71 (12.3)	397 (13.8)	468 (13.5)
Other	27 (4.7)	50 (1.7)	77 (2.2)
White	477 (83.0)	2437 (84.5)	2914 (84.2)
Ethnicity, n (%)			
N-Miss	18	55	73
Hispanic	6 (1.1)	32 (1.1)	38 (1.1)
Non-Hispanic	552 (98.9)	2797 (98.9)	3349 (98.9)
Insurance type, n (%)			
N-Miss	13	226	239
Medicaid	72 (12.8)	362 (13.6)	434 (13.5)
Medicare	235 (41.7)	1478 (55.6)	1713 (53.2)
Other insurance	32 (5.7)	208 (7.8)	240 (7.5)
Private	213 (37.8)	482 (18.1)	695 (21.6)
Uninsured	11 (2.0)	128 (4.8)	139 (4.3)
SEER stage, n (%)			
Localized	435 (75.5)	1143 (39.6)	1578 (45.6)
Regional	92 (16.0)	855 (29.6)	947 (27.4)
Distant	27 (4.7)	575 (19.9)	602 (17.4)
Unstaged	22 (3.8)	311 (10.8)	333 (9.6)
Marital status, n (%)			
N-Miss	52	480	532
Divorced	58 (11.1)	269 (11.2)	327 (11.2)
Married	326 (62.2)	1125 (46.8)	1451 (49.6)
Separated	5 (1.0)	34 (1.4)	39 (1.3)
Single	89 (17.0)	406 (16.9)	495 (16.9)
Unknown	18 (3.4)	322 (13.4)	340 (11.6)
Widowed	28 (5.3)	248 (10.3)	276 (9.4)
County type, n (%)			
Urban	465 (80.7)	2309 (80.1)	2774 (80.2)
Rural, urban adjacent	93 (16.1)	466 (16.2)	559 (16.2)
Rural, not urban adjacent	18 (3.1)	109 (3.8)	127 (3.7)
County SDI			
Mean (SD)	49.0 (29.5)	49.2 (28.2)	49.2 (28.4)
Median (Q1, Q3)	50.0 (23.0, 69.0)	50.0 (23.0, 69.0)	50.0 (23.0, 69.0)
Range	1.0–90.0	1.0–90.0	1.0–90.0
County SDI by quartile, n (%)			
Q1	160 (27.8)	774 (26.8)	934 (27.0)
Q2	137 (23.8)	681 (23.6)	818 (23.6)
Q3	141 (24.5)	754 (26.1)	895 (25.9)
Q4	138 (24.0)	675 (23.4)	813 (23.5)
Neighborhood SDI			
Mean (SD)	50.1 (29.4)	55.8 (28.7)	54.9 (28.9)
Median (Q1, Q3)	50.0 (23.0, 78.0)	57.0 (31.0, 83.0)	56.0 (29.0, 82.0)
Range	1.0–100.0	1.0–100.0	1.0–100.0
Neighborhood SDI by quartile, n (%)			
Q1	183 (31.8)	684 (23.7)	867 (25.1)
Q2	143 (24.8)	728 (25.2)	871 (25.2)
Q3	134 (23.3)	735 (25.5)	869 (25.1)
Q4	116 (20.1)	737 (25.6)	853 (24.7)
Neighborhood FPL 100 score			
Mean (SD)	52.4 (28.7)	57.7 (28.2)	56.8 (28.3)
Median (Q1, Q3)	52.0 (28.0, 79.0)	57.0 (36.0, 83.2)	57.0 (35.0, 83.0)
Range	1.0–100.0	1.0–100.0	1.0–100.0
Neighborhood FPL 100 score by quartile			
Q1	183 (31.8)	707 (24.5)	890 (25.7)
Q2	147 (25.5)	736 (25.5)	883 (25.5)
Q3	131 (22.7)	720 (25.0)	851 (24.6)
Q4	115 (20.0)	721 (25.0)	836 (24.2)

Abbreviations: FPL, federal poverty level; SEER, Surveillance, Epidemiology, and End Results; SDI, social deprivation index.

**TABLE 2:** Demographic, clinical characteristics, neighborhood, and individual social determinants of health in patients with cholangiocarcinoma by survival status

	Alive (N=68)	Deceased (N=713)	Total (N=781)
Age at diagnosis			
Mean (SD)	62.5 (11.4)	68.0 (12.1)	67.5 (12.1)
Median (Q1, Q3)	64.0 (56.0, 69.0)	68.0 (59.0, 77.0)	68.0 (59.0, 76.0)
Range	25.0–87.0	33.0–97.0	25.0–97.0
Sex, n (%)			
Female	38 (55.9)	372 (52.2)	410 (52.5)
Male	30 (44.1)	341 (47.8)	371 (47.5)
Race, n (%)			
Black	3 (4.4)	47 (6.6)	50 (6.4)
White	65 (95.6)	666 (93.4)	731 (93.6)
Ethnicity, n (%)			
N-Miss	2	8	10
Hispanic	0	3 (0.4)	3 (0.4)
Non-Hispanic	66 (100.0)	702 (99.6)	768 (99.6)
Insurance, n (%)			
N-Miss	3	43	46
Medicaid	3 (4.6)	35 (5.2)	38 (5.2)
Medicare	28 (43.1)	412 (61.5)	440 (59.9)
Other insurance	3 (4.6)	53 (7.9)	56 (7.6)
Private	28 (43.1)	154 (23.0)	182 (24.8)
Uninsured	3 (4.6)	16 (2.4)	19 (2.6)
SEER stage			
Localized	30 (44.1)	167 (23.4)	197 (25.2)
Regional	19 (27.9)	197 (27.6)	216 (27.7)
Distant	16 (23.5)	270 (37.9)	286 (36.6)
Unstaged	3 (4.4)	79 (11.1)	82 (10.5)
Marital status, n (%)			
N-Miss	18	99	117
Divorced	5 (10.0)	77 (12.5)	82 (12.3)
Married (including common law)	33 (66.0)	304 (49.5)	337 (50.8)
Separated	0	3 (0.5)	3 (0.5)
Single (never married)	5 (10.0)	65 (10.6)	70 (10.5)
Unknown	2 (4.0)	59 (9.6)	61 (9.2)
Widowed	5 (10.0)	106 (17.3)	111 (16.7)
County type, n (%)			
Urban	52 (76.5)	560 (78.5)	612 (78.4)
Rural—urban adjacent	13 (19.1)	121 (17.0)	134 (17.2)
Rural—not urban adjacent	3 (4.4)	32 (4.5)	35 (4.5)
County SDI			
Mean (SD)	41.3 (27.8)	44.8 (26.4)	44.5 (26.5)
Median (Q1, Q3)	40.5 (22.0, 58.0)	50.0 (22.0, 69.0)	47.0 (22.0, 69.0)
Range	2.0–90.0	1.0–90.0	1.0–90.0
County SDI by quartile, n (%)			
Q1	20 (29.4)	195 (27.3)	215 (27.5)
Q2	25 (36.8)	151 (21.2)	176 (22.5)
Q3	11 (16.2)	255 (35.8)	266 (34.1)
Q4	12 (17.6)	112 (15.7)	124 (15.9)
Neighborhood SDI			
Mean (SD)	44.4 (28.1)	48.5 (28.0)	48.2 (28.0)
Median (Q1, Q3)	38.0 (21.8, 67.5)	49.0 (24.0, 72.0)	48.0 (24.0, 72.0)
Range	3.0–96.0	1.0–100.0	1.0–100.0
Neighborhood SDI by quartile, n (%)			
Q1	18 (26.5)	176 (24.7)	194 (24.8)
Q2	20 (29.4)	177 (24.8)	197 (25.2)
Q3	15 (22.1)	189 (26.5)	204 (26.1)
Q4	15 (22.1)	171 (24.0)	186 (23.8)
Neighborhood FPL 100 score			
Mean (SD)	49.2 (26.9)	51.1 (27.6)	50.9 (27.5)
Median (Q1, Q3)	47.5 (27.5, 69.0)	50.0 (29.0, 74.0)	50.0 (29.0, 74.0)
Range	1.0–98.0	1.0–100.0	1.0–100.0
Neighborhood FPL 100 score by quartile, n (%)			
Q1	18 (26.5)	177 (24.8)	195 (25.0)
Q2	17 (25.0)	177 (24.8)	194 (24.8)
Q3	20 (29.4)	182 (25.5)	202 (25.9)
Q4	13 (19.1)	177 (24.8)	190 (24.3)

Abbreviations: FPL, federal poverty level; SEER, Surveillance, Epidemiology, and End Results; SDI, social deprivation index.

### Individual SDOH and rurality by neighborhood social deprivation quartiles

For individuals with HCC, the median neighborhood SDI was 56.0 (IQR: 29.0–82.0) (100 indicating the most deprived) (Table 1), and for quartiles 1 and 4, it was 17.0 (IQR: 10.0–24.0) and 92.0 (IQR: 87.0–95.0), respectively (Table 3). For individuals with CCA, the median neighborhood SDI was 48 (IQR: 24.0–72.0) (Table 2), and for quartiles 1 and 4, it was 13.0 (IQR: 8.0–19.0) and 86.0 (IQR: 79.0–93.0), respectively (Table 4).

**TABLE 3:** Demographic, clinical characteristics, neighborhood, and individual social determinants of health in patients with HCC by neighborhood SDI quartile

	Q1 (N=867)	Q2 (N=871)	Q3 (N=869)	Q4 (N=853)	*p*
Age at diagnosis	—	—	—	—	<0.0001
Mean (SD)	66.7 (10.8)	66.2 (11.2)	65.1 (11.6)	62.6 (10.3)	—
Median (Q1, Q3)	66.0 (59.0, 75.0)	65.0 (59.0, 74.0)	63.0 (57.0, 73.0)	61.0 (56.0, 68.0)	—
Range	24.0–94.0	19.0–97.0	24.0–98.0	23.0–96.0	—
Sex, n (%)	—	—	—	—	0.6463
Female	206 (23.8)	230 (26.4)	218 (25.1)	217 (25.4)	—
Male	661 (76.2)	641 (73.6)	651 (74.9)	636 (74.6)	—
Race, n (%)	—	—	—	—	<0.0001
N-Miss	1	0	0	0	—
Black	31 (3.6)	42 (4.8)	115 (13.2)	280 (32.8)	—
Other	36 (4.2)	15 (1.7)	15 (1.7)	11 (1.3)	—
White	799 (92.3)	814 (93.5)	739 (85.0)	562 (65.9)	—
Ethnicity, n (%)	—	—	—	—	0.1242
N-Miss	11	15	16	31	—
Hispanic	7 (0.8)	5 (0.6)	13 (1.5)	13 (1.6)	—
Non-Hispanic	849 (99.2)	851 (99.4)	840 (98.5)	809 (98.4)	—
Insurance, n (%)	—	—	—	—	<0.0001
N-Miss	48	55	62	74	—
Medicaid	62 (7.6)	82 (10.0)	111 (13.8)	179 (23.0)	—
Medicare	466 (56.9)	460 (56.4)	423 (52.4)	364 (46.7)	—
Other insurance	62 (7.6)	56 (6.9)	52 (6.4)	70 (9.0)	—
Private	214 (26.1)	177 (21.7)	184 (22.8)	120 (15.4)	—
Uninsured	15 (1.8)	41 (5.0)	37 (4.6)	46 (5.9)	—
SEER stage, n (%)	—	—	—	—	0.3104
Localized	418 (48.2)	408 (46.8)	386 (44.4)	366 (42.9)	—
Regional	227 (26.2)	231 (26.5)	242 (27.8)	247 (29.0)	—
Distant	142 (16.4)	154 (17.7)	143 (16.5)	163 (19.1)	—
Unstaged	80 (9.2)	78 (9.0)	98 (11.3)	77 (9.0)	—
Marital, n (%)	—	—	—	—	<0.0001
N-Miss	100	135	139	158	—
Divorced	57 (7.4)	73 (9.9)	82 (11.2)	115 (16.5)	—
Married (including common law)	484 (63.1)	382 (51.9)	341 (46.7)	244 (35.1)	—
Separated	2 (0.3)	7 (1.0)	19 (2.6)	11 (1.6)	—
Single (Never Married)	82 (10.7)	106 (14.4)	121 (16.6)	186 (26.8)	—
Unknown	71 (9.3)	91 (12.4)	93 (12.7)	85 (12.2)	—
Widowed	71 (9.3)	77 (10.5)	74 (10.1)	54 (7.8)	—
County type, n (%)					
Urban	758 (87.4)	593 (68.1)	624 (71.8)	799 (93.7)	—
Rural-urban adjacent	93 (10.7)	224 (25.7)	205 (23.6)	37 (4.3)	—
Rural-not urban adjacent	16 (1.8)	54 (6.2)	40 (4.6)	17 (2.0)	—
County SDI					
Mean (SD)	35.5 (29.2)	39.9 (24.8)	51.4 (25.0)	70.3 (20.7)	—
Median (Q1, Q3)	28.0 (9.0, 56.0)	40.0 (20.0, 58.0)	51.0 (34.0, 69.0)	69.0 (51.0, 90.0)	—
Range	1.0–90.0	1.0–90.0	1.0–90.0	14.0–90.0	—
County SDI by quartile, n (%)					
Q1	405 (46.7)	332 (38.1)	171 (19.7)	26 (3.0)	—
Q2	153 (17.6)	227 (26.1)	263 (30.3)	175 (20.5)	—
Q3	207 (23.9)	216 (24.8)	238 (27.4)	234 (27.4)	—
Q4	102 (11.8)	96 (11.0)	197 (22.7)	418 (49.0)	—
Neighborhood SDI					
Mean (SD)	16.6 (8.3)	43.0 (8.1)	69.2 (7.6)	91.2 (4.7)	—
Median (Q1, Q3)	17.0 (10.0, 24.0)	43.0 (36.0, 51.0)	69.0 (63.0, 76.0)	92.0 (87.0, 95.0)	—
Range	1.0–29.0	30.0–56.0	57.0–82.0	83.0–100.0	—
Neighborhood FPL 100 score					
Mean (SD)	21.7 (13.0)	46.4 (13.4)	68.4 (12.5)	91.4 (6.8)	—
Median (Q1, Q3)	20.0 (11.0, 30.5)	46.0 (37.0, 55.0)	70.0 (58.0, 78.0)	93.0 (88.0, 97.0)	—
Range	1.0–55.0	7.0–93.0	34.0–97.0	66.0–100.0	—
Neighborhood FPL 100 score by quartile					
Q1	714 (82.4)	174 (20.0)	2 (0.2)	0	—
Q2	153 (17.6)	529 (60.7)	201 (23.1)	0	—
Q3	0	166 (19.1)	575 (66.2)	110 (12.9)	—
Q4	0	2 (0.2)	91 (10.5)	743 (87.1)	—

Abbreviations: FPL, federal poverty level; SEER, Surveillance, Epidemiology, and End Results; SDI, social deprivation index.

**TABLE 4:** Demographic, clinical characteristics, neighborhood, and individual social determinants of health in patients with cholangiocarcinoma by neighborhood SDI quartile

	Q1 (N=194)	Q2 (N=197)	Q3 (N=204)	Q4 (N=186)	*p*
Age at diagnosis	—	—	—	—	0.5547
Mean (SD)	66.9 (12.1)	67.6 (12.3)	68.4 (11.7)	66.9 (12.3)	—
Median (Q1, Q3)	68.0 (57.0, 75.8)	68.0 (60.0, 76.0)	68.0 (60.8, 77.0)	66.0 (58.0, 76.0)	—
Range	37.0–97.0	35.0–97.0	39.0–96.0	25.0–92.0	—
Sex, n (%)	—	—	—	—	0.0318
Female	87 (44.8)	101 (51.3)	111 (54.4)	111 (59.7)	—
Male	107 (55.2)	96 (48.7)	93 (45.6)	75 (40.3)	—
Race, n (%)	—	—	—	—	<0.0001
Black	1 (0.5)	3 (1.5)	12 (5.9)	34 (18.3)	—
White	193 (99.5)	194 (98.5)	192 (94.1)	152 (81.7)	—
Ethnicity, n (%)	—	—	—	—	0.2616
N-Miss	1	1	2	6	—
Hispanic	0	0	1 (0.5)	2 (1.1)	—
Non-Hispanic	193 (100.0)	196 (100.0)	201 (99.5)	178 (98.9)	—
Insurance, n (%)	—	—	—	—	0.0004
N-Miss	5	10	14	17	—
Medicaid	4 (2.1)	3 (1.6)	10 (5.3)	21 (12.4)	—
Medicare	111 (58.7)	112 (59.9)	116 (61.1)	101 (59.8)	—
Other Insurance	17 (9.0)	15 (8.0)	14 (7.4)	10 (5.9)	—
Private	55 (29.1)	52 (27.8)	45 (23.7)	30 (17.8)	—
Uninsured	2 (1.1)	5 (2.7)	5 (2.6)	7 (4.1)	—
SEER stage	—	—	—	—	0.3556
Localized	50 (25.8)	54 (27.4)	46 (22.5)	47 (25.3)	—
Regional	60 (30.9)	56 (28.4)	52 (25.5)	48 (25.8)	—
Distant	70 (36.1)	63 (32.0)	87 (42.6)	66 (35.5)	—
Unstaged	14 (7.2)	24 (12.2)	19 (9.3)	25 (13.4)	—
Marital status, n (%)	—	—	—	—	<0.0001
N-Miss	26	34	31	26	—
Divorced	13 (7.7)	19 (11.7)	21 (12.1)	29 (18.1)	—
Married (including common law)	112 (66.7)	94 (57.7)	76 (43.9)	55 (34.4)	—
Separated	0	1 (0.6)	1 (0.6)	1 (0.6)	—
Single (never married)	13 (7.7)	11 (6.7)	23 (13.3)	23 (14.4)	—
Unknown	14 (8.3)	13 (8.0)	15 (8.7)	19 (11.9)	—
Widowed	16 (9.5)	25 (15.3)	37 (21.4)	33 (20.6)	—
County type					
Urban	168 (86.6)	146 (74.1)	146 (71.6)	152 (81.7)	—
Rural-urban adjacent	20 (10.3)	43 (21.8)	46 (22.5)	25 (13.4)	—
Rural—not urban adjacent	6 (3.1)	8 (4.1)	12 (5.9)	9 (4.8)	—
County SDI					
Mean (SD)	36.2 (27.5)	36.3 (24.3)	45.5 (23.7)	60.8 (22.9)	—
Median (Q1, Q3)	36.0 (12.0, 53.0)	28.0 (14.0, 58.0)	46.5 (23.0, 69.0)	59.0 (50.0, 90.0)	—
Range	1.0–90.0	1.0–90.0	2.0–90.0	9.0–90.0	—
County SDI by quartile, n (%)					
Q1	74 (38.1)	81 (41.1)	46 (22.5)	14 (7.5)	—
Q2	44 (22.7)	42 (21.3)	61 (29.9)	29 (15.6)	—
Q3	57 (29.4)	63 (32.0)	66 (32.4)	80 (43.0)	—
Q4	19 (9.8)	11 (5.6)	31 (15.2)	63 (33.9)	—
Neighborhood SDI					
Mean (SD)	13.1 (6.3)	34.6 (7.5)	59.9 (7.4)	86.1 (7.6)	—
Median (Q1, Q3)	13.0 (8.0, 19.0)	34.0 (28.0, 40.0)	59.0 (53.0, 67.0)	86.0 (79.0, 93.0)	—
Range	1.0–23.0	24.0–48.0	49.0–72.0	73.0–100.0	—
FPL 100 score					
Mean (SD)	18.8 (11.2)	39.4 (13.6)	60.8 (13.0)	85.8 (11.0)	—
Median (Q1, Q3)	17.0 (10.0, 26.8)	40.0 (31.0, 47.0)	61.0 (52.0, 70.0)	88.5 (80.0, 93.0)	—
Range	1.0–45.0	6.0–74.0	28.0–98.0	44.0–100.0	—
FPL 100 score by quartile, n (%)					
Q1	157 (80.9)	37 (18.8)	1 (0.5)	0	—
Q2	37 (19.1)	119 (60.4)	37 (18.1)	1 (0.5)	—
Q3	0	41 (20.8)	139 (68.1)	22 (11.8)	—
Q4	0	0	27 (13.2)	163 (87.6)	—

Abbreviations: FPL, federal poverty level; SEER, Surveillance, Epidemiology, and End Results; SDI, social deprivation index.

For HCC, individuals residing in quartile 4 SDI neighborhoods (most deprived) were more likely to be Black, never married, or insured by Medicaid than individuals residing in quartile 1 neighborhoods (*p*-values <0.0001) (Table 3). There was no difference in cancer stage between the SDI quartiles (*p*=0.310) (Table 3).

For CCA, individuals residing in quartile 4 SDI neighborhoods (most deprived) were more likely to be women (*p*=0.032), Black (*p*<0.0001), never married (*p*<0.0001), or insured by Medicaid (*p*=0.0004) than individuals residing in quartile 1 SDI neighborhoods (least deprived) (Table 4). There was no detected difference in cancer stage between the SDI quartiles (*p*=0.356) (Table 4).

### Mortality

#### HCC

From the Cox proportional hazard model, in HCC, mortality was associated with older age of diagnosis, male sex, and more advanced SEER stage (Table 5). Regarding the SDOH, individuals residing in quartile 4 SDI neighborhoods (most deprived) had higher mortality than individuals residing in quartile 1 SDI neighborhoods (HR: 1.14, 95% CI, 1.003–1.30, *p*=0.045) (Table 5). Mortality was associated with being never married compared with married (HR: 1.31, 95% CI, 1.15–1.48, *p*<0.0001) and being uninsured (HR: 1.64, 95% CI, 1.30–2.07, *p*<0.0001) or having Medicare (HR: 1.14, 95% CI, 1.001–1.30, *p*=0.048) compared with private insurance. County SDI quartile was not significantly associated with mortality (Table 5).

**TABLE 5:** Cox proportional hazard models for time from diagnosis to death in patients with HCC including SDI quartile

Effect	Univariable HR	*p*	Multivariable HR	95% HR confidence limits		*p* (*p*<0.05)
Race (overall *p*)	—	0.0048	—	—	—	0.0785
Race—Black vs. White	0.91	0.0691	0.88	0.77	1.01	0.0726
Race—other vs. White	0.67	0.0049	0.79	0.56	1.09	0.1529
Sex—male vs. female	1.14	0.0026	1.20	1.08	1.33	0.0005^a^
Ethnicity—Hispanic vs. non-Hispanic	1.43	0.0441	1.36	0.90	2.04	0.1446
Age at diagnosis	1.01	<0.0001	1.02	1.01	1.02	<0.0001^a^
SEER summary stage (overall *p*-value)	—	<0.0001	—	—	—	<0.0001^a^
SEER stage—distant vs. localized	3.23	<0.0001	3.36	2.98	3.78	<0.0001^a^
SEER stage—regional vs. localized	2.02	<0.0001	2.02	1.83	2.24	<0.0001^a^
SEER stage—unstaged vs. localized	2.32	<0.0001	2.09	1.75	2.50	<0.0001^a^
Marital status (overall *p*-value)	—	<0.0001	—	—	—	0.0003^a^
Marital—divorced vs. married	1.05	0.4557	1.14	0.99	1.32	0.0699
Marital—separated vs. married	1.13	0.4794	1.25	0.87	1.79	0.2349
Marital—never married vs. married	1.20	0.0019	1.31	1.15	1.48	<0.0001^a^
Marital—unknown vs. married	1.58	<0.0001	1.24	1.06	1.45	0.0083^a^
Marital—widowed vs. married	1.27	0.0006	1.22	1.05	1.42	0.0098^a^
Insurance status (overall *p*-value)	—	<0.0001	—	—	—	0.0009^a^
Insurance—Medicaid vs. private	1.20	0.0091	1.08	0.93	1.26	0.3176
Insurance—Medicare vs. private	1.42	<0.0001	1.14	1.001	1.30	0.0480^a^
Insurance—other insurance vs. private	1.41	<0.0001	1.16	0.97	1.39	0.1107
Insurance—uninsured vs. private	2.36	<0.0001	1.64	1.30	2.07	<0.0001^a^
Area type (overall *p*-value)	—	0.1490	—	—	—	—
Urban vs. rural—not urban adjacent	1.12	0.2583	—	—	—	—
Rural—urban adjacent vs. rural—not urban adjacent	1.21	0.0800	—	—	—	—
County SDI quartile (overall *p*-value)	—	0.0753	—	—	—	—
County SDI quartile—Q2 vs. Q1	1.02	0.7641	—	—	—	—
County SDI quartile—Q3 vs. Q1	1.04	0.4291	—	—	—	—
County SDI quartile—Q4 vs. Q1	0.91	0.0825	—	—	—	—
Neighborhood SDI quartile (overall *p*-value)	—	0.0520	—	—	—	0.2448
Neighborhood SDI quartile—Q2 vs. Q1	1.10	0.0809	1.04	0.93	1.18	0.4774
Neighborhood SDI quartile—Q3 vs. Q1	1.12	0.0302	1.07	0.95	1.21	0.2626
Neighborhood SDI quartile—Q4 vs. Q1	1.15	0.0091	1.14	1.003	1.30	0.0447^a^

Abbreviations: SEER, Surveillance, Epidemiology, and End Results; SDI, social deprivation index.

^a^ Significant *p* value.

When substituting SDI with neighborhood income below the FPL in the multivariable Cox regression model, living in a neighborhood within the fourth quartile for FPL was associated with mortality (HR: 1.14, 95% CI, 1.01–1.30, *p*=0.042) (Supplemental Table 1, http://links.lww.com/HC9/A137).

#### Cholangiocarcinoma

From the Cox proportional hazard model, mortality in CCA was associated with older age at diagnosis and more advanced SEER stage (Table 6). Regarding the SDOH, being never married was associated with mortality (HR: 1.34, 95% CI, 1.008–1.79, *p*=0.0499). Neither county nor neighborhood SDI was not associated with time to death (Table 6).

**TABLE 6:** Cox proportional hazard models for time from diagnosis to death in patients with cholangiocarcinoma including SDI quartile

Effect	Univariable HR	*p*	Multivariable HR	95% HR confidence limits		*p* (*p*<0.05)
Race—Black vs. White	1.10	0.5533	1.02	0.71	1.47	0.9186
Sex—male vs. female	0.99	0.8964	1.05	0.88	1.25	0.6203
Ethnicity—Hispanic vs. non-Hispanic	0.68	0.4973	—	—	—	—
Age at diagnosis	1.02	<0.0001	1.03	1.02	1.04	<0.0001^a^
SEER stage (overall *p*-value)	—	<0.0001	—	—	—	<0.0001^a^
SEER stage—distant vs. localized	2.12	<0.0001	2.22	1.77	2.77	<0.0001^a^
SEER stage—regional vs. localized	1.44	0.0006	1.59	1.26	2.02	0.0001^a^
SEER stage—unstaged vs. localized	2.19	<0.0001	1.86	1.35	2.56	0.0001^a^
Marital status (overall *p*-value)	—	0.0192	—	—	—	0.0912
Marital—divorced vs. married	1.27	0.0637	1.32	1.01	1.73	0.0439^a^
Marital—separated vs. married	0.49	0.2192	0.43	0.13	1.37	0.1527
Marital—never married vs. married	1.30	0.0599	1.34	1.008	1.79	0.0499^a^
Marital—unknown vs. married	1.39	0.0207	1.24	0.85	1.79	0.2602
Marital—widowed vs. married	1.31	0.0179	1.08	0.83	1.39	0.5765
Insurance status (overall *p*-value)	—	0.0322	—	—	—	0.0420^a^
Insurance—Medicaid vs. private	1.13	0.5242	0.89	0.58	1.36	0.5823
Insurance—Medicare vs. private	1.35	0.0016	0.78	0.60	1.01	0.0571
Insurance—other insurance vs. private	1.23	0.1964	1.17	0.83	1.64	0.3756
Insurance—uninsured vs. private	1.39	0.2108	1.50	0.87	2.60	0.1451
Area type (overall *p*-value)	—	0.3681	—	—	—	—
Urban vs. rural—not urban adjacent	0.79	0.1991	—	—	—	—
Rural—urban adjacent vs. rural	0.85	0.4171	—	—	—	—
County SDI quartile (overall *p*-value)	—	0.3399	—	—	—	—
County SDI quartile—Q2 vs. Q1	0.95	0.6676	—	—	—	—
County SDI quartile—Q3 vs. Q1	1.11	0.2624	—	—	—	—
County SDI quartile—Q4 vs. Q1	1.14	0.2881	—	—	—	—
Neighborhood SDI quartile (overall *p*-value)	—	0.1736	—	—	—	0.5065
Neighborhood SDI quartile—Q2 vs. Q1	1.02	0.8417	1.11	0.88	1.40	0.3916
Neighborhood SDI quartile—Q3 vs. Q1	1.22	0.0637	1.17	0.92	1.47	0.1956
Neighborhood SDI quartile—Q4 vs. Q1	1.17	0.1559	1.19	0.93	1.52	0.1763

Abbreviations: SEER, Surveillance, Epidemiology, and End Results; SDI, social deprivation index.

^a^ Significant *p* value.

In the univariable analysis, neighborhood income below the FPL quartile was also significantly associated with time to death (Supplemental Table 2, http://links.lww.com/HC9/A137).

#### Geographic mortality clusters in HCC

**FIGURE 1:**
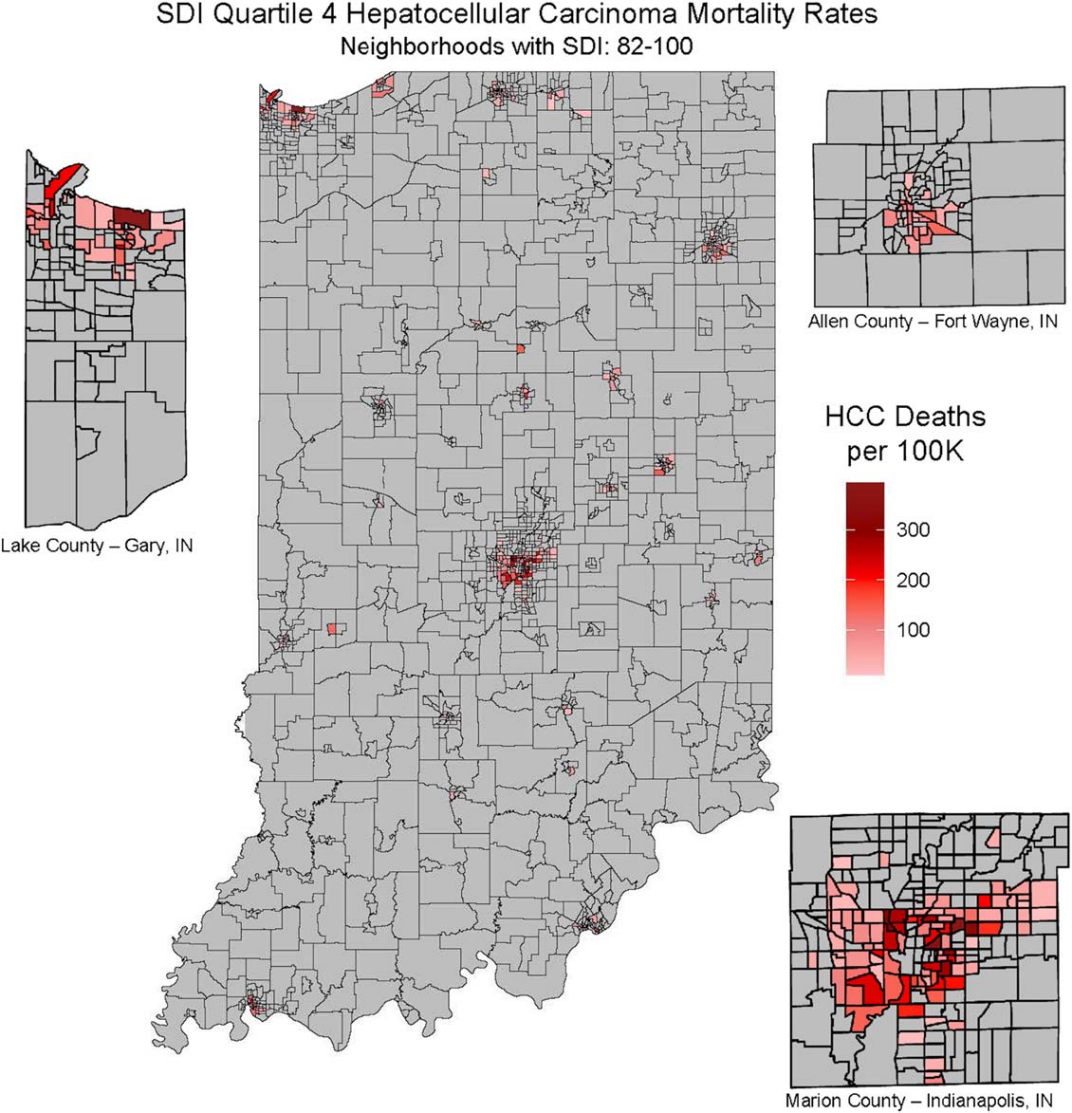
SDI quartile 4 deaths in patients with HCC. The number of deaths in quartile 4 SDI neighborhoods per 100,000 thousand residents. Abbreviations: SDI, social deprivation index.

Over the course of the study period 83.4% of individuals with HCC were deceased. Mortality from HCC clustered in high SDI census tracts, largely localized in urban centers (Indianapolis, Gary, and Fort Wayne, IN) (Figure 1). Clustering in CCA was not explored given SDI was not associated with mortality.

#### Cumulative burden of SDOH in HCC

**FIGURE 2:**
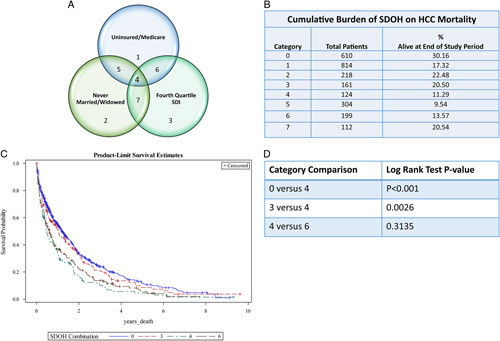
*Note*: individuals without the SDOH included in the venn diagram are in category. The cumulative burden of social determinants of health on HCC survival. (A) The SDOH that were associated with mortality. (B) Proportion alive at the end of the study period in the categories identified in Panel A. (C) Survival curves for selected categories. (D) Log-rank testing between selected categories. Abbreviations: SDI, social deprivation index; SDOH, social determinants of health.

To determine the cumulative burden of SDOH on HCC mortality, patients with 1 SDOH that was significantly associated with mortality [uninsured/Medicare (category 1), never married/widowed (category 2), or fourth quartile SDI (category 3)] were compared with patients with 2 SDOH (categories 5, 6, and 7) or 3 SDOH (category 4) (Figure 2A). Among 2542 patients with complete data on SDOH 23.9% had none of the 3 SDOH associated with mortality and were grouped in category 0. By the end of the study period, there was a significant difference in survival between categories 0 and 4 (*p*<0.0001) and between categories 3 and 4 (*p*=0.003) (Figure 2).

## DISCUSSION

In the last 5 years, HCC mortality has begun declining among US adults.^24^ However, disparities in survival are known to still exist by race, ethnicity, insurance status, and geography.^25^ Furthermore, area disadvantage has been associated with mortality in other cancers.^26^ Yet, the association between area and individual-level SDOH and mortality has not been evaluated in HCC and CCA. Here, we demonstrate that in HCC, both area and individual SDOH matters. However, in CCA where treatment options are limited and the mortality rates are quite high, area-level SDOH may be less important. Finally, although rural geography was not significantly associated with mortality, high SDI urban mortality “hotspots” were identified in HCC.

Individuals with HCC living in a neighborhood in the fourth quartile for SDI were 14% more likely to be deceased than those living in neighborhoods in the first quartile. Income below the FPL was also found to be associated with mortality. Area disadvantage has been associated with incidence and mortality in other cancers.^27^,^28^ Specifically, in a recent SEER database analysis, area deprivation was associated with worse survival among patients with nonmetastatic breast, prostate, lung, and colorectal cancers after accounting for individual SES.^26^ The role of neighborhood disadvantage, until now, had not been evaluated in HCC or CCA. Notably however, census-tract level poverty was also associated with mortality in HCC.^13^ Therefore, although a composite measure may be more sensitive, independent measures of neighborhood poverty may be sufficient for identifying cohorts to target for interventions.

In addition to area deprivation, having Medicare or being uninsured, as well as having a marital status of never being married or widowed, were associated with mortality in HCC. This is consistent with what has previously been seen in the literature. In a retrospective SEER Medicare-linked database study when stratified by neighborhood poverty, Black patients living in high-poverty neighborhoods had worse survival (HR: 1.13; 95% CI, 1.02–1.25).^14^ In an analysis by Wong and colleagues, patients from rural regions and lower-income households had more advanced tumor stage at diagnosis and significantly higher HCC mortality. In CCA, never married marital status was the only SDOH associated with mortality. There have been very few studies looking at SDOH in CCA.^16^,^29^ In a study exploring individual income and CCA outcomes, low income was associated with poor survival (< $37,999 vs. ≥ $63,000 annually; HR: 1.07, 95% CI, 1.01–1.14, *p* = 0.032).^29^ However, this analysis did not include marital status, a surrogate marker for social support. Notably, SDI was not associated with time to death in CCA suggesting that in some high mortality cancers, stage is the strongest predictor of survival.

Rurality and SDI had evidence of collinearity in our analysis, suggesting a strong relationship between neighborhood deprivation and rural-urban residence in this cohort. Mortality from neighborhoods in the fourth quartile of SDI was mostly clustered into 3 urban counties in HCC, including Marion and Lake County, which contain Indianapolis and Gary, IN, respectively. In 2019, Gary, IN, was ranked worse economically than any city in the US based on the percentage of people in the workforce, median household income, percentage of people without health insurance, median commute times, and the number of people living in poverty.^30^ A SEER database study found that patients who lived in large metropolitan areas of over 1 million people had a better overall survival than other cohorts.^13^ None of the 3 urban hotspots we identified has populations over 1 million residents. Therefore, these findings may be consistent with previous work.

Finally, we explored the cumulative burden of the SDOH on mortality in HCC and found that there was indeed an association. Multiple SDOH has been shown to increase mortality in adults with diabetes and chronic kidney disease; however, its effect on HCC mortality has not been previously explored.^31^,^32^

### Steps toward cancer control and prevention

Taken together, area measures of deprivation may allow the identification of neighborhoods with the highest levels of disadvantage to better allocate time, effort, and resources. Combined with the relevant individual SDOH targeted evidence-based cancer control policies and interventions can be implemented in the populations with the highest risk for mortality. Community-based cancer control interventions that have demonstrated efficacy include culturally and demographically tailored cancer screening programs, informed decision-making tools, and community-based genomic, obesity, and sun safety programs.^33^ Successful interventions to reduce disparities are often multilevel and resource intense.^34^ In our own research group, these data are being used to identify the highest risk groups for intervention development and testing. Furthermore, there are a growing number of international (UK and New Zealand) and US regional programs (Massachusetts and Hennepin Health Center in Minnesota Medicaid Demonstration Project) that are using a blend of individual SDOH and neighborhood measures of deprivation to adjust payments for primary and social services.^11^ For other states to follow this lead, tumor-specific, target communities need to be identified.

Our study included over 4000 patients spanning over 9 years making it the largest study to explore the role of SDI while controlling for individual determinants in these cancer types. Indiana, with its large rural population, uneven distribution of area wealth, and high burden of chronic liver disease, is uniquely suited to explore these questions. Our population was over 12% Black and nearly approximates the proportion of Black Americans living in the US but included fewer individuals of Hispanic and Asian ancestry than the US population. Interestingly, Black race was not associated with mortality in this cohort as it has been in other data sets.^14^,^25^ This likely reflects the high mortality rate in both Black and White patients with HCC experienced in Indiana. The CIs for the association with some of the SDOH and mortality were near one. However, our cumulative burden subgroup analysis suggests that indeed these factors do impact mortality. This analysis, however, assumes that each SDOH is equally weighted. Further work is needed to understand the relative weight of each SDOH on cancer mortality. On the basis of our previous work that includes some patients from this cohort, we hypothesize that the mechanism by which the SDOH impacts mortality is through access to treatments like liver transplantation.^35^ Prospective studies are needed to further clarify this relationship. Finally, for this analysis, we did not have data on liver disease severity and comorbidity, which may also impact mortality. However, in an analysis that did include measures of comorbidity, neighborhood poverty remained independently associated with mortality.^14^ We hypothesize that the strength of the association would also remain significant.

In conclusion, both area-level and individual-level SDOH are associated with mortality in HCC. These associations are not as clear in CCA. If we are to develop effective cancer control and prevention interventions that improve health equity, both area and individual determinants of health should be considered.

## Supplementary Material

## References

[1] Chakraborty E Sarkar D. Emerging therapies for hepatocellular carcinoma (HCC). Cancers (Basel). 2022;14. doi:10.3390/cancers14112798 PMC9179883 35681776

[2] Llovet JM Kelley RK Villanueva A Singal AG Pikarsky E Roayaie S. Hepatocellular carcinoma. Nat Rev Dis Primers. 2021;7:6. 33479224 10.1038/s41572-020-00240-3 PMC13537433

[3] Shah C Mramba LK Bishnoi R Bejjanki H Chhatrala HS Chandana SR. Survival differences among patients with hepatocellular carcinoma based on the stage of disease and therapy received: pre and post sorafenib era. J Gastrointest Oncol. 2017;8:789–98. 29184682 10.21037/jgo.2017.06.16 PMC5674257

[4] Shroff RT Kennedy EB Bachini M Bekaii-Saab T Crane C Edeline J. Adjuvant therapy for resected biliary tract cancer: ASCO clinical practice guideline. J Clin Oncol. 2019;37:1015–27. 30856044 10.1200/JCO.18.02178

[5] Salgia R Mendiratta V. The multidisciplinary management of hepatocellular carcinoma. Clin Liver Dis. 2021;17:405–8. 10.1002/cld.1068 PMC8340356 34386204

[6] Alcaraz KI Wiedt TL Daniels EC Yabroff KR Guerra CE Wender RC. Understanding and addressing social determinants to advance cancer health equity in the United States: a blueprint for practice, research, and policy. CA: Cancer J Clin. 2020;70:31–46. 31661164 10.3322/caac.21586

[7] Nephew LD Aitcheson G Iyengar M. The impact of racial disparities on liver disease access and outcomes. Curr Treatm Opt Gastroenterol. 2022;20. doi:10.1007/s11938-022-00390-1

[8] Social Determinats of Health. https://health.gov/healthypeople/priority-areas/social-determinants-health

[9] Shrider EA Kollar M Chen F Semega J. US Current Population Reports, Income and Poverty in the United States: 2020. Census Bureau; 2020.

[10] Butler DC Petterson S Phillips RL Bazemore AW. Measures of social deprivation that predict health care access and need within a rational area of primary care service delivery. Health Serv Res. 2013;48(2 pt 1):539–59. 22816561 10.1111/j.1475-6773.2012.01449.x PMC3626349

[11] Huffstetler AN Phillips RL. Payment structures that support social care integration with clinical care: social deprivation indices and novel payment models. Am J Prev Med. 2019;57(6, suppl 1):S82–S88. 31753283 10.1016/j.amepre.2019.07.011

[12] Dobis EABLJ Zhalnin AV Kumar I Dimensions of Indiana poverty. https://www.ibrc.indiana.edu/ibr/2019/fall/article1.html

[13] Wong RJ Kim D Ahmed A Singal AK. Patients with hepatocellular carcinoma from more rural and lower-income households have more advanced tumor stage at diagnosis and significantly higher mortality. Cancer. 2021;127:45–55. 33103243 10.1002/cncr.33211

[14] Wagle NS Park S Washburn D Ohsfeldt RL Rich NE Singal AG. Racial, ethnic, and socioeconomic disparities in curative treatment receipt and survival in hepatocellular carcinoma. Hepatol Commun. 2022;6:1186–97. 34796703 10.1002/hep4.1863 PMC9035560

[15] Oluyomi AO El-Serag HB Olayode A Thrift AP. Neighborhood-level factors contribute to disparities in hepatocellular carcinoma incidence in Texas. Clin Gastroenterol Hepatol. 2022; S1542-3565(22)00711-X. doi: 10.1016/j.cgh.2022.06.031 PMC9898456 35933074

[16] Zhu MX Li Y. The correlations between socioeconomic status and intrahepatic cholangiocarcinoma in the United States: a population-based study. Transl Cancer Res. 2020;9:4931–42. 35117855 10.21037/tcr-20-2506 PMC8798916

[17] Shi L Stevens GD. Vulnerability and unmet health care needs. The influence of multiple risk factors. J Gen Intern Med. 2005;20:148–54. 15836548 10.1111/j.1525-1497.2005.40136.x PMC1490048

[18] Pinquart M Duberstein PR. Associations of social networks with cancer mortality: a meta-analysis. Crit Rev Oncol Hematol. 2010;75:122–37; (In eng). 19604706 10.1016/j.critrevonc.2009.06.003 PMC2910231

[19] Liang Z Nau C Xie F Vogel R Chen W. The application of community-based information from the American Community Survey in a large integrated health care organization. Perm J. 2020;25:1–3. 10.7812/TPP/20.010 PMC8803254 33635758

[20] U.S Department of Agriculture. Rural-Urban Commuting Area (RUCA) Codes.https://www.ers.usda.gov/data-products/rural-urban-commuting-area-codes/

[21] Mohamed KA Ghabril M Desai A Orman E Patidar KR Holden J. Neighborhood poverty is associated with failure to be waitlisted and death during liver transplantation evaluation. Liver Transpl. 2022;28:1441–53. 35389564 10.1002/lt.26473 PMC9545792

[22] Administration HRaS. Defining Rural Population. https://www.hrsa.gov/rural-health/about-us/what-is-rural

[23] Data Quality AaIBSRP. Summary stage 2018 general coding instructions. https://seer.cancer.gov/tools/ssm/2018-Summary-Stage-Manual.pdf

[24] Lee YT Wang JJ Luu M Noureddin M Kosari K Agopian VG. The mortality and overall survival trends of primary liver cancer in the United States. J Natl Cancer Inst. 2021;113:1531–41. 34010422 10.1093/jnci/djab079 PMC8562972

[25] Ajayi F Jan J Singal AG Rich NE. Racial and sex disparities in hepatocellular carcinoma in the USA. Curr Hepatol Rep. 2020;19:462–9. 33828937 10.1007/s11901-020-00554-6 PMC8020839

[26] Cheng E Soulos PR Irwin ML Cespedes Feliciano EM Presley CJ Fuchs CS. Neighborhood and individual socioeconomic disadvantage and survival among patients with nonmetastatic common cancers. JAMA Netw Open. 2021;4:e2139593. 34919133 10.1001/jamanetworkopen.2021.39593 PMC8683967

[27] Babatunde OA Zahnd WE Eberth JM Lawson AB Adams SA Boakye EA. Association between neighborhood social deprivation and stage at diagnosis among breast cancer patients in South Carolina. Int J Environ Res Public Health. 2021;18. doi:10.3390/ijerph182211824 PMC8625868 34831579

[28] Sorice KA Fang CY Wiese D Ortiz A Chen Y Henry KA. Systematic review of neighborhood socioeconomic indices studied across the cancer control continuum. Cancer Medicine. 2022;11:2125–44. 35166051 10.1002/cam4.4601 PMC9119356

[29] Uhlig J Sellers CM Cha C Khan SA Lacy J Stein SM. Intrahepatic cholangiocarcinoma: socioeconomic discrepancies, contemporary treatmentapproaches and survival trends from the national cancer database. Ann Surg Oncol. 2019;26:1993–2000; (In eng). 30693451 10.1245/s10434-019-07175-4

[30] PJaW A. The 50 most miserable cities in America, based on census data. https://www.businessinsider.com/most-miserable-cities-in-the-united-states-based-on-data-2019-9

[31] Ozieh MN Garacci E Walker RJ Palatnik A Egede LE. The cumulative impact of social determinants of health factors on mortality in adults with diabetes and chronic kidney disease. BMC Nephrol. 2021;22:76. 33639878 10.1186/s12882-021-02277-2 PMC7916298

[32] Pinheiro LC Reshetnyak E Akinyemiju T Phillips E Safford MM. Social determinants of health and cancer mortality in the Reasons for Geographic and Racial Differences in Stroke (REGARDS) cohort study. Cancer. 2022;128:122–30. 34478162 10.1002/cncr.33894 PMC9301452

[33] National Cancer Institute. Evidence-Based Cancer Control Programs (EBCCP). https://ebccp.cancercontrol.cancer.gov/index.do

[34] Naylor K Ward J Polite BN. Interventions to improve care related to colorectal cancer among racial and ethnic minorities: a systematic review. J Gen Intern Med. 2012;27:1033–46. 22798214 10.1007/s11606-012-2044-2 PMC3403155

[35] Dakhoul L Gawrieh S Jones KR Ghabril M McShane C Orman E. Racial disparities in liver transplantation for hepatocellular carcinoma are not explained by differences in comorbidities, liver disease severity, or tumor burden. Hepatol Commun. 2019;3:52–62. 30619994 10.1002/hep4.1277 PMC6312653

