# Supplementary figures and images for: Social determinants of health impact mortality from HCC and cholangiocarcinoma: a population-based cohort study
Source: Hepatol Commun. 2023 Feb 9;7(3):e0058. doi: 10.1097/HC9.0000000000000058 (PMC9916098; doi:10.1097/HC9.0000000000000058)

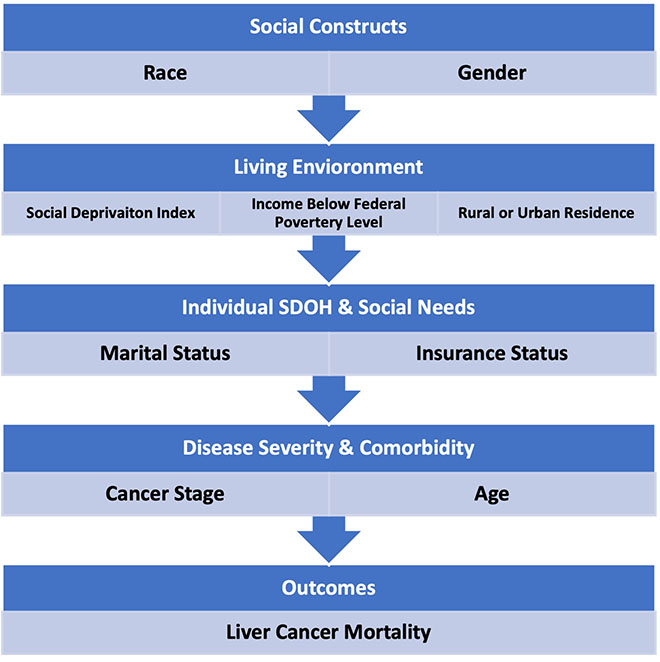

Supplement: Supplementary file 1 [file hc9-7-e0058-s001.jpg]
