# Supplementary material for: Social determinants of health impact mortality from HCC and cholangiocarcinoma: a population-based cohort study
Source: Hepatol Commun. 2023 Feb 9;7(3):e0058. doi: 10.1097/HC9.0000000000000058 (PMC9916098; doi:10.1097/HC9.0000000000000058)
Supplement: Supplementary file 2 [file hc9-7-e0058-s002.docx]

## Table 1. Demographic, clinical characteristics, neighborhood and individual SDOH in patients with hepatocellular carcinoma by survival status

|  | Alive (N=576) | Deceased (N=2884) | Total (N=3460) |
| --- | --- | --- | --- |
| **Age at Diagnosis** |  |  |  |
| Mean (SD) | 62.0 (10.2) | 65.8 (11.2) | 65.2 (11.1) |
| Median (Q1, Q3) | 62.0 (56.8, 68.0) | 64.0 (58.0, 74.0) | 64.0 (57.0, 73.0) |
| Range | 20.0 - 90.0 | 19.0 - 98.0 | 19.0 - 98.0 |
| **Sex** |  |  |  |
| Female | 160 (27.8%) | 711 (24.7%) | 871 (25.2%) |
| Male | 416 (72.2%) | 2173 (75.3%) | 2589 (74.8%) |
| **Race** |  |  |  |
| N-Miss | 1 | 0 | 1 |
| Black | 71 (12.3%) | 397 (13.8%) | 468 (13.5%) |
| Other | 27 (4.7%) | 50 (1.7%) | 77 (2.2%) |
| White | 477 (83.0%) | 2437 (84.5%) | 2914 (84.2%) |
| **Ethnicity** |  |  |  |
| N-Miss | 18 | 55 | 73 |
| Hispanic | 6 (1.1%) | 32 (1.1%) | 38 (1.1%) |
| Non-Hispanic | 552 (98.9%) | 2797 (98.9%) | 3349 (98.9%) |
| **Insurance Type** |  |  |  |
| N-Miss | 13 | 226 | 239 |
| Medicaid | 72 (12.8%) | 362 (13.6%) | 434 (13.5%) |
| Medicare | 235 (41.7%) | 1478 (55.6%) | 1713 (53.2%) |
| Other Insurance | 32 (5.7%) | 208 (7.8%) | 240 (7.5%) |
| Private | 213 (37.8%) | 482 (18.1%) | 695 (21.6%) |
| Uninsured | 11 (2.0%) | 128 (4.8%) | 139 (4.3%) |
| **SEER Stage** |  |  |  |
| Localized | 435 (75.5%) | 1143 (39.6%) | 1578 (45.6%) |
| Regional | 92 (16.0%) | 855 (29.6%) | 947 (27.4%) |
| Distant | 27 (4.7%) | 575 (19.9%) | 602 (17.4%) |
| Unstaged | 22 (3.8%) | 311 (10.8%) | 333 (9.6%) |
| **Marital Status** |  |  |  |
| N-Miss | 52 | 480 | 532 |
| Divorced | 58 (11.1%) | 269 (11.2%) | 327 (11.2%) |
| Married | 326 (62.2%) | 1125 (46.8%) | 1451 (49.6%) |
| Separated | 5 (1.0%) | 34 (1.4%) | 39 (1.3%) |
| Single | 89 (17.0%) | 406 (16.9%) | 495 (16.9%) |
| Unknown | 18 (3.4%) | 322 (13.4%) | 340 (11.6%) |
| Widowed | 28 (5.3%) | 248 (10.3%) | 276 (9.4%) |
| **County Type** |  |  |  |
| Urban | 465 (80.7%) | 2309 (80.1%) | 2774 (80.2%) |
| Rural – Urban Adjacent | 93 (16.1%) | 466 (16.2%) | 559 (16.2%) |
| Rural – Not Urban Adjacent | 18 (3.1%) | 109 (3.8%) | 127 (3.7%) |
| **County SDI** |  |  |  |
| Mean (SD) | 49.0 (29.5) | 49.2 (28.2) | 49.2 (28.4) |
| Median (Q1, Q3) | 50.0 (23.0, 69.0) | 50.0 (23.0, 69.0) | 50.0 (23.0, 69.0) |
| Range | 1.0 - 90.0 | 1.0 - 90.0 | 1.0 - 90.0 |
| **County SDI by Quartile** |  |  |  |
| Q1 | 160 (27.8%) | 774 (26.8%) | 934 (27.0%) |
| Q2 | 137 (23.8%) | 681 (23.6%) | 818 (23.6%) |
| Q3 | 141 (24.5%) | 754 (26.1%) | 895 (25.9%) |
| Q4 | 138 (24.0%) | 675 (23.4%) | 813 (23.5%) |
| **Neighborhood SDI** |  |  |  |
| Mean (SD) | 50.1 (29.4) | 55.8 (28.7) | 54.9 (28.9) |
| Median (Q1, Q3) | 50.0 (23.0, 78.0) | 57.0 (31.0, 83.0) | 56.0 (29.0, 82.0) |
| Range | 1.0 - 100.0 | 1.0 - 100.0 | 1.0 - 100.0 |
| **Neighborhood SDI by Quartile** |  |  |  |
| Q1 | 183 (31.8%) | 684 (23.7%) | 867 (25.1%) |
| Q2 | 143 (24.8%) | 728 (25.2%) | 871 (25.2%) |
| Q3 | 134 (23.3%) | 735 (25.5%) | 869 (25.1%) |
| Q4 | 116 (20.1%) | 737 (25.6%) | 853 (24.7%) |
| **Neighborhood FPL 100 score** |  |  |  |
| Mean (SD) | 52.4 (28.7) | 57.7 (28.2) | 56.8 (28.3) |
| Median (Q1, Q3) | 52.0 (28.0, 79.0) | 57.0 (36.0, 83.2) | 57.0 (35.0, 83.0) |
| Range | 1.0 - 100.0 | 1.0 - 100.0 | 1.0 - 100.0 |
| **Neighborhood FPL 100 score by Quartile** |  |  |  |
| Q1 | 183 (31.8%) | 707 (24.5%) | 890 (25.7%) |
| Q2 | 147 (25.5%) | 736 (25.5%) | 883 (25.5%) |
| Q3 | 131 (22.7%) | 720 (25.0%) | 851 (24.6%) |
| Q4 | 115 (20.0%) | 721 (25.0%) | 836 (24.2%) |

## Table 2. Demographic, clinical characteristics, neighborhood and individual SDOH in patients with cholangiocarcinoma by survival status

|  | Alive (N=68) | Deceased (N=713) | Total (N=781) |
| --- | --- | --- | --- |
| **Age at Diagnosis** |  |  |  |
| Mean (SD) | 62.5 (11.4) | 68.0 (12.1) | 67.5 (12.1) |
| Median (Q1, Q3) | 64.0 (56.0, 69.0) | 68.0 (59.0, 77.0) | 68.0 (59.0, 76.0) |
| Range | 25.0 - 87.0 | 33.0 - 97.0 | 25.0 - 97.0 |
| **Sex** |  |  |  |
| Female | 38 (55.9%) | 372 (52.2%) | 410 (52.5%) |
| Male | 30 (44.1%) | 341 (47.8%) | 371 (47.5%) |
| **Race** |  |  |  |
| Black | 3 (4.4%) | 47 (6.6%) | 50 (6.4%) |
| White | 65 (95.6%) | 666 (93.4%) | 731 (93.6%) |
| **Ethnicity** |  |  |  |
| N-Miss | 2 | 8 | 10 |
| Hispanic | 0 (0.0%) | 3 (0.4%) | 3 (0.4%) |
| Non-Hispanic | 66 (100.0%) | 702 (99.6%) | 768 (99.6%) |
| **Insurance** |  |  |  |
| N-Miss | 3 | 43 | 46 |
| Medicaid | 3 (4.6%) | 35 (5.2%) | 38 (5.2%) |
| Medicare | 28 (43.1%) | 412 (61.5%) | 440 (59.9%) |
| Other Insurance | 3 (4.6%) | 53 (7.9%) | 56 (7.6%) |
| Private | 28 (43.1%) | 154 (23.0%) | 182 (24.8%) |
| Uninsured | 3 (4.6%) | 16 (2.4%) | 19 (2.6%) |
| **SEER Stage** |  |  |  |
| Localized | 30 (44.1%) | 167 (23.4%) | 197 (25.2%) |
| Regional | 19 (27.9%) | 197 (27.6%) | 216 (27.7%) |
| Distant | 16 (23.5%) | 270 (37.9%) | 286 (36.6%) |
| Unstaged | 3 (4.4%) | 79 (11.1%) | 82 (10.5%) |
| **Marital Status** |  |  |  |
| N-Miss | 18 | 99 | 117 |
| Divorced | 5 (10.0%) | 77 (12.5%) | 82 (12.3%) |
| Married (Including Common Law) | 33 (66.0%) | 304 (49.5%) | 337 (50.8%) |
| Separated | 0 (0.0%) | 3 (0.5%) | 3 (0.5%) |
| Single (Never Married) | 5 (10.0%) | 65 (10.6%) | 70 (10.5%) |
| Unknown | 2 (4.0%) | 59 (9.6%) | 61 (9.2%) |
| Widowed | 5 (10.0%) | 106 (17.3%) | 111 (16.7%) |
| **County Type** |  |  |  |
| Urban | 52 (76.5%) | 560 (78.5%) | 612 (78.4%) |
| Rural - Urban Adjacent | 13 (19.1%) | 121 (17.0%) | 134 (17.2%) |
| Rural - Not Urban Adjacent | 3 (4.4%) | 32 (4.5%) | 35 (4.5%) |
| **County SDI** |  |  |  |
| Mean (SD) | 41.3 (27.8) | 44.8 (26.4) | 44.5 (26.5) |
| Median (Q1, Q3) | 40.5 (22.0, 58.0) | 50.0 (22.0, 69.0) | 47.0 (22.0, 69.0) |
| Range | 2.0 - 90.0 | 1.0 - 90.0 | 1.0 - 90.0 |
| **County SDI by Quartile** |  |  |  |
| Q1 | 20 (29.4%) | 195 (27.3%) | 215 (27.5%) |
| Q2 | 25 (36.8%) | 151 (21.2%) | 176 (22.5%) |
| Q3 | 11 (16.2%) | 255 (35.8%) | 266 (34.1%) |
| Q4 | 12 (17.6%) | 112 (15.7%) | 124 (15.9%) |
| **Neighborhood SDI** |  |  |  |
| Mean (SD) | 44.4 (28.1) | 48.5 (28.0) | 48.2 (28.0) |
| Median (Q1, Q3) | 38.0 (21.8, 67.5) | 49.0 (24.0, 72.0) | 48.0 (24.0, 72.0) |
| Range | 3.0 - 96.0 | 1.0 - 100.0 | 1.0 - 100.0 |
| **Neighborhood SDI by Quartile** |  |  |  |
| Q1 | 18 (26.5%) | 176 (24.7%) | 194 (24.8%) |
| Q2 | 20 (29.4%) | 177 (24.8%) | 197 (25.2%) |
| Q3 | 15 (22.1%) | 189 (26.5%) | 204 (26.1%) |
| Q4 | 15 (22.1%) | 171 (24.0%) | 186 (23.8%) |
| **Neighborhood FPL 100 score** |  |  |  |
| Mean (SD) | 49.2 (26.9) | 51.1 (27.6) | 50.9 (27.5) |
| Median (Q1, Q3) | 47.5 (27.5, 69.0) | 50.0 (29.0, 74.0) | 50.0 (29.0, 74.0) |
| Range | 1.0 - 98.0 | 1.0 - 100.0 | 1.0 - 100.0 |
| **Neighborhood FPL 100 score by Quartile** |  |  |  |
| Q1 | 18 (26.5%) | 177 (24.8%) | 195 (25.0%) |
| Q2 | 17 (25.0%) | 177 (24.8%) | 194 (24.8%) |
| Q3 | 20 (29.4%) | 182 (25.5%) | 202 (25.9%) |
| Q4 | 13 (19.1%) | 177 (24.8%) | 190 (24.3%) |

**Table 3.** Demographic, clinical characteristics, neighborhood and individual SDOH in patients with **hepatocellular carcinoma** by neighborhood **SDI Quartile**

|  | Q1 (N=867) | Q2 (N=871) | Q3 (N=869) | Q4 (N=853) | p-value |
| --- | --- | --- | --- | --- | --- |
| **Age at Diagnosis** |  |  |  |  | <.0001 |
| Mean (SD) | 66.7 (10.8) | 66.2 (11.2) | 65.1 (11.6) | 62.6 (10.3) |  |
| Median (Q1, Q3) | 66.0 (59.0, 75.0) | 65.0 (59.0, 74.0) | 63.0 (57.0, 73.0) | 61.0 (56.0, 68.0) |  |
| Range | 24.0 - 94.0 | 19.0 - 97.0 | 24.0 - 98.0 | 23.0 - 96.0 |  |
| **Sex** |  |  |  |  | 0.6463 |
| Female | 206 (23.8%) | 230 (26.4%) | 218 (25.1%) | 217 (25.4%) |  |
| Male | 661 (76.2%) | 641 (73.6%) | 651 (74.9%) | 636 (74.6%) |  |
| **Race** |  |  |  |  | <.0001 |
| N-Miss | 1 | 0 | 0 | 0 |  |
| Black | 31 (3.6%) | 42 (4.8%) | 115 (13.2%) | 280 (32.8%) |  |
| Other | 36 (4.2%) | 15 (1.7%) | 15 (1.7%) | 11 (1.3%) |  |
| White | 799 (92.3%) | 814 (93.5%) | 739 (85.0%) | 562 (65.9%) |  |
| **Ethnicity** |  |  |  |  | .1242 |
| N-Miss | 11 | 15 | 16 | 31 |  |
| Hispanic | 7 (0.8%) | 5 (0.6%) | 13 (1.5%) | 13 (1.6%) |  |
| Non-Hispanic | 849 (99.2%) | 851 (99.4%) | 840 (98.5%) | 809 (98.4%) |  |
| **Insurance** |  |  |  |  | <.0001 |
| N-Miss | 48 | 55 | 62 | 74 |  |
| Medicaid | 62 (7.6%) | 82 (10.0%) | 111 (13.8%) | 179 (23.0%) |  |
| Medicare | 466 (56.9%) | 460 (56.4%) | 423 (52.4%) | 364 (46.7%) |  |
| Other Insurance | 62 (7.6%) | 56 (6.9%) | 52 (6.4%) | 70 (9.0%) |  |
| Private | 214 (26.1%) | 177 (21.7%) | 184 (22.8%) | 120 (15.4%) |  |
| Uninsured | 15 (1.8%) | 41 (5.0%) | 37 (4.6%) | 46 (5.9%) |  |
| **SEER Stage** |  |  |  |  | 0.3104 |
| Localized | 418 (48.2%) | 408 (46.8%) | 386 (44.4%) | 366 (42.9%) |  |
| Regional | 227 (26.2%) | 231 (26.5%) | 242 (27.8%) | 247 (29.0%) |  |
| Distant | 142 (16.4%) | 154 (17.7%) | 143 (16.5%) | 163 (19.1%) |  |
| Unstaged | 80 (9.2%) | 78 (9.0%) | 98 (11.3%) | 77 (9.0%) |  |
| **Marital** |  |  |  |  | <.0001 |
| N-Miss | 100 | 135 | 139 | 158 |  |
| Divorced | 57 (7.4%) | 73 (9.9%) | 82 (11.2%) | 115 (16.5%) |  |
| Married (Including Common Law) | 484 (63.1%) | 382 (51.9%) | 341 (46.7%) | 244 (35.1%) |  |
| Separated | 2 (0.3%) | 7 (1.0%) | 19 (2.6%) | 11 (1.6%) |  |
| Single (Never Married) | 82 (10.7%) | 106 (14.4%) | 121 (16.6%) | 186 (26.8%) |  |
| Unknown | 71 (9.3%) | 91 (12.4%) | 93 (12.7%) | 85 (12.2%) |  |
| Widowed | 71 (9.3%) | 77 (10.5%) | 74 (10.1%) | 54 (7.8%) |  |
| **County Type** |  |  |  |  |  |
| Urban | 758 (87.4%) | 593 (68.1%) | 624 (71.8%) | 799 (93.7%) |  |
| Rural - Urban Adjacent | 93 (10.7%) | 224 (25.7%) | 205 (23.6%) | 37 (4.3%) |  |
| Rural - Not Urban Adjacent | 16 (1.8%) | 54 (6.2%) | 40 (4.6%) | 17 (2.0%) |  |
| **County SDI** |  |  |  |  |  |
| Mean (SD) | 35.5 (29.2) | 39.9 (24.8) | 51.4 (25.0) | 70.3 (20.7) |  |
| Median (Q1, Q3) | 28.0 (9.0, 56.0) | 40.0 (20.0, 58.0) | 51.0 (34.0, 69.0) | 69.0 (51.0, 90.0) |  |
| Range | 1.0 - 90.0 | 1.0 - 90.0 | 1.0 - 90.0 | 14.0 - 90.0 |  |
| **County SDI by Quartile** |  |  |  |  |  |
| Q1 | 405 (46.7%) | 332 (38.1%) | 171 (19.7%) | 26 (3.0%) |  |
| Q2 | 153 (17.6%) | 227 (26.1%) | 263 (30.3%) | 175 (20.5%) |  |
| Q3 | 207 (23.9%) | 216 (24.8%) | 238 (27.4%) | 234 (27.4%) |  |
| Q4 | 102 (11.8%) | 96 (11.0%) | 197 (22.7%) | 418 (49.0%) |  |
| **Neighborhood SDI** |  |  |  |  |  |
| Mean (SD) | 16.6 (8.3) | 43.0 (8.1) | 69.2 (7.6) | 91.2 (4.7) |  |
| Median (Q1, Q3) | 17.0 (10.0, 24.0) | 43.0 (36.0, 51.0) | 69.0 (63.0, 76.0) | 92.0 (87.0, 95.0) |  |
| Range | 1.0 - 29.0 | 30.0 - 56.0 | 57.0 - 82.0 | 83.0 - 100.0 |  |
| **Neighborhood FPL 100 score** |  |  |  |  |  |
| Mean (SD) | 21.7 (13.0) | 46.4 (13.4) | 68.4 (12.5) | 91.4 (6.8) |  |
| Median (Q1, Q3) | 20.0 (11.0, 30.5) | 46.0 (37.0, 55.0) | 70.0 (58.0, 78.0) | 93.0 (88.0, 97.0) |  |
| Range | 1.0 - 55.0 | 7.0 - 93.0 | 34.0 - 97.0 | 66.0 - 100.0 |  |
| **Neighborhood FPL 100 score by Quartile** |  |  |  |  |  |
| Q1 | 714 (82.4%) | 174 (20.0%) | 2 (0.2%) | 0 (0.0%) |  |
| Q2 | 153 (17.6%) | 529 (60.7%) | 201 (23.1%) | 0 (0.0%) |  |
| Q3 | 0 (0.0%) | 166 (19.1%) | 575 (66.2%) | 110 (12.9%) |  |
| Q4 | 0 (0.0%) | 2 (0.2%) | 91 (10.5%) | 743 (87.1%) |  |
|  |  |  |  |  |  |
|  |  |  |  |  |  |
|  |  |  |  |  |  |
|  |  |  |  |  |  |
|  |  |  |  |  |  |
|  |  |  |  |  |  |
|  |  |  |  |  |  |
|  |  |  |  |  |  |
|  |  |  |  |  |  |

**Table 4.** Demographic, clinical characteristics, neighborhood and individual SDOH in patients with **cholangiocarcinoma** by neighborhood **SDI Quartile**

|  | Q1 (N=194) | Q2 (N=197) | Q3 (N=204) | Q4 (N=186) | p-value |
| --- | --- | --- | --- | --- | --- |
| **Age at Diagnosis** |  |  |  |  | 0.5547 |
| Mean (SD) | 66.9 (12.1) | 67.6 (12.3) | 68.4 (11.7) | 66.9 (12.3) |  |
| Median (Q1, Q3) | 68.0 (57.0, 75.8) | 68.0 (60.0, 76.0) | 68.0 (60.8, 77.0) | 66.0 (58.0, 76.0) |  |
| Range | 37.0 - 97.0 | 35.0 - 97.0 | 39.0 - 96.0 | 25.0 - 92.0 |  |
| **Sex** |  |  |  |  | 0.0318 |
| Female | 87 (44.8%) | 101 (51.3%) | 111 (54.4%) | 111 (59.7%) |  |
| Male | 107 (55.2%) | 96 (48.7%) | 93 (45.6%) | 75 (40.3%) |  |
| **Race** |  |  |  |  | <.0001 |
| Black | 1 (0.5%) | 3 (1.5%) | 12 (5.9%) | 34 (18.3%) |  |
| White | 193 (99.5%) | 194 (98.5%) | 192 (94.1%) | 152 (81.7%) |  |
| **Ethnicity** |  |  |  |  | 0.2616 |
| N-Miss | 1 | 1 | 2 | 6 |  |
| Hispanic | 0 (0.0%) | 0 (0.0%) | 1 (0.5%) | 2 (1.1%) |  |
| Non-Hispanic | 193 (100.0%) | 196 (100.0%) | 201 (99.5%) | 178 (98.9%) |  |
| **Insurance** |  |  |  |  | 0.0004 |
| N-Miss | 5 | 10 | 14 | 17 |  |
| Medicaid | 4 (2.1%) | 3 (1.6%) | 10 (5.3%) | 21 (12.4%) |  |
| Medicare | 111 (58.7%) | 112 (59.9%) | 116 (61.1%) | 101 (59.8%) |  |
| Other Insurance | 17 (9.0%) | 15 (8.0%) | 14 (7.4%) | 10 (5.9%) |  |
| Private | 55 (29.1%) | 52 (27.8%) | 45 (23.7%) | 30 (17.8%) |  |
| Uninsured | 2 (1.1%) | 5 (2.7%) | 5 (2.6%) | 7 (4.1%) |  |
| **SEER Stage** |  |  |  |  | 0.3556 |
| Localized | 50 (25.8%) | 54 (27.4%) | 46 (22.5%) | 47 (25.3%) |  |
| Regional | 60 (30.9%) | 56 (28.4%) | 52 (25.5%) | 48 (25.8%) |  |
| Distant | 70 (36.1%) | 63 (32.0%) | 87 (42.6%) | 66 (35.5%) |  |
| Unstaged | 14 (7.2%) | 24 (12.2%) | 19 (9.3%) | 25 (13.4%) |  |
| **Marital Status** |  |  |  |  | <.0001 |
| N-Miss | 26 | 34 | 31 | 26 |  |
| Divorced | 13 (7.7%) | 19 (11.7%) | 21 (12.1%) | 29 (18.1%) |  |
| Married (Including Common Law) | 112 (66.7%) | 94 (57.7%) | 76 (43.9%) | 55 (34.4%) |  |
| Separated | 0 (0.0%) | 1 (0.6%) | 1 (0.6%) | 1 (0.6%) |  |
| Single (Never Married) | 13 (7.7%) | 11 (6.7%) | 23 (13.3%) | 23 (14.4%) |  |
| Unknown | 14 (8.3%) | 13 (8.0%) | 15 (8.7%) | 19 (11.9%) |  |
| Widowed | 16 (9.5%) | 25 (15.3%) | 37 (21.4%) | 33 (20.6%) |  |
| **County Type** |  |  |  |  |  |
| Urban | 168 (86.6%) | 146 (74.1%) | 146 (71.6%) | 152 (81.7%) |  |
| Rural - Urban Adjacent | 20 (10.3%) | 43 (21.8%) | 46 (22.5%) | 25 (13.4%) |  |
| Rural - Not Urban Adjacent | 6 (3.1%) | 8 (4.1%) | 12 (5.9%) | 9 (4.8%) |  |
| **County SDI** |  |  |  |  |  |
| Mean (SD) | 36.2 (27.5) | 36.3 (24.3) | 45.5 (23.7) | 60.8 (22.9) |  |
| Median (Q1, Q3) | 36.0 (12.0, 53.0) | 28.0 (14.0, 58.0) | 46.5 (23.0, 69.0) | 59.0 (50.0, 90.0) |  |
| Range | 1.0 - 90.0 | 1.0 - 90.0 | 2.0 - 90.0 | 9.0 - 90.0 |  |
| **County SDI by Quartile** |  |  |  |  |  |
| Q1 | 74 (38.1%) | 81 (41.1%) | 46 (22.5%) | 14 (7.5%) |  |
| Q2 | 44 (22.7%) | 42 (21.3%) | 61 (29.9%) | 29 (15.6%) |  |
| Q3 | 57 (29.4%) | 63 (32.0%) | 66 (32.4%) | 80 (43.0%) |  |
| Q4 | 19 (9.8%) | 11 (5.6%) | 31 (15.2%) | 63 (33.9%) |  |
| **Neighborhood SDI** |  |  |  |  |  |
| Mean (SD) | 13.1 (6.3) | 34.6 (7.5) | 59.9 (7.4) | 86.1 (7.6) |  |
| Median (Q1, Q3) | 13.0 (8.0, 19.0) | 34.0 (28.0, 40.0) | 59.0 (53.0, 67.0) | 86.0 (79.0, 93.0) |  |
| Range | 1.0 - 23.0 | 24.0 - 48.0 | 49.0 - 72.0 | 73.0 - 100.0 |  |
| **FPL 100 score** |  |  |  |  |  |
| Mean (SD) | 18.8 (11.2) | 39.4 (13.6) | 60.8 (13.0) | 85.8 (11.0) |  |
| Median (Q1, Q3) | 17.0 (10.0, 26.8) | 40.0 (31.0, 47.0) | 61.0 (52.0, 70.0) | 88.5 (80.0, 93.0) |  |
| Range | 1.0 - 45.0 | 6.0 - 74.0 | 28.0 - 98.0 | 44.0 - 100.0 |  |
| **FPL 100 score by Quartile** |  |  |  |  |  |
| Q1 | 157 (80.9%) | 37 (18.8%) | 1 (0.5%) | 0 (0.0%) |  |
| Q2 | 37 (19.1%) | 119 (60.4%) | 37 (18.1%) | 1 (0.5%) |  |
| Q3 | 0 (0.0%) | 41 (20.8%) | 139 (68.1%) | 22 (11.8%) |  |
| Q4 | 0 (0.0%) | 0 (0.0%) | 27 (13.2%) | 163 (87.6%) |  |

## Table 5 Cox proportional hazard models for time from diagnosis to death in patients with hepatocellular carcinoma including SDI quartile

| Effect | Univariable  Hazard Ratio | p-value | Multivariable  Hazard Ratio | 95% Hazard Ratio Confidence Limits | | p-value | (p<.05) |
| --- | --- | --- | --- | --- | --- | --- | --- |
| Race (Overall p-value) | -- | 0.0048 | -- | -- | -- | 0.0785 |  |
| Race – Black vs White | 0.91 | 0.0691 | 0.88 | 0.77 | 1.01 | 0.0726 |  |
| Race – Other vs White | 0.67 | 0.0049 | 0.79 | 0.56 | 1.09 | 0.1529 |  |
| Sex – Male vs Female | 1.14 | 0.0026 | 1.20 | 1.08 | 1.33 | 0.0005 | * |
| Ethnicity – Hispanic vs Non Hispanic | 1.43 | 0.0441 | 1.36 | 0.90 | 2.04 | 0.1446 |  |
| Age at Diagnosis | 1.01 | <.0001 | 1.02 | 1.01 | 1.02 | <.0001 | * |
| SEER Summary Stage (Overall p-value) | -- | <.0001 | -- | -- | -- | <.0001 | * |
| SEER Stage – Distant vs Localized | 3.23 | <.0001 | 3.36 | 2.98 | 3.78 | <.0001 | * |
| SEER Stage – Regional vs Localized | 2.02 | <.0001 | 2.02 | 1.83 | 2.24 | <.0001 | * |
| SEER Stage – Unstaged vs Localized | 2.32 | <.0001 | 2.09 | 1.75 | 2.50 | <.0001 | * |
| Marital Status (Overall p-value) | -- | <.0001 | -- | -- | -- | 0.0003 | * |
| Marital – Divorced vs Married | 1.05 | 0.4557 | 1.14 | 0.99 | 1.32 | 0.0699 |  |
| Marital – Separated vs Married | 1.13 | 0.4794 | 1.25 | 0.87 | 1.79 | 0.2349 |  |
| Marital – Never Married vs Married | 1.20 | 0.0019 | 1.31 | 1.15 | 1.48 | <.0001 | * |
| Marital – Unknown vs Married | 1.58 | <.0001 | 1.24 | 1.06 | 1.45 | 0.0083 | * |
| Marital – Widowed vs Married | 1.27 | 0.0006 | 1.22 | 1.05 | 1.42 | 0.0098 | * |
| Insurance Status (Overall p-value) | -- | <.0001 | -- | -- | -- | 0.0009 | * |
| Insurance – Medicaid vs Private | 1.20 | 0.0091 | 1.08 | 0.93 | 1.26 | 0.3176 |  |
| Insurance – Medicare vs Private | 1.42 | <.0001 | 1.14 | 1.001 | 1.30 | 0.0480 | * |
| Insurance – Other Insurance vs Private | 1.41 | <.0001 | 1.16 | 0.97 | 1.39 | 0.1107 |  |
| Insurance – Uninsured vs Private | 2.36 | <.0001 | 1.64 | 1.30 | 2.07 | <.0001 | * |
| Area Type (Overall p-value) | -- | 0.1490 |  |  |  |  |  |
| Urban vs. Rural – Not Urban Adjacent | 1.12 | 0.2583 |  |  |  |  |  |
| Rural – Urban Adjacent vs. Rural – Not Urban Adjacent | 1.21 | 0.0800 |  |  |  |  |  |
| County SDI Quartile (Overall p-value) | -- | 0.0753 |  |  |  |  |  |
| County SDI Quartile – Q2 vs Q1 | 1.02 | 0.7641 |  |  |  |  |  |
| County SDI Quartile – Q3 vs Q1 | 1.04 | 0.4291 |  |  |  |  |  |
| County SDI Quartile – Q4 vs Q1 | 0.91 | 0.0825 |  |  |  |  |  |
| Neighborhood SDI Quartile (Overall p-value) | -- | 0.0520 | -- | -- | -- | 0.2448 |  |
| Neighborhood SDI Quartile – Q2 vs Q1 | 1.10 | 0.0809 | 1.04 | 0.93 | 1.18 | 0.4774 |  |
| Neighborhood SDI Quartile – Q3 vs Q1 | 1.12 | 0.0302 | 1.07 | 0.95 | 1.21 | 0.2626 |  |
| Neighborhood SDI Quartile – Q4 vs Q1 | 1.15 | 0.0091 | 1.14 | 1.003 | 1.30 | 0.0447 | * |
|  | | | | | | | |

## Table 6 Cox proportional hazard models for time from diagnosis to death in patients with cholangiocarcinoma including SDI quartile

| Effect | Univariable Hazard Ratio | P-value | Multivariable Hazard Ratio | 95% Hazard Ratio Confidence Limits | | p-value | (p<.05) |
| --- | --- | --- | --- | --- | --- | --- | --- |
| Race – Black vs White | 1.10 | 0.5533 | 1.02 | 0.71 | 1.47 | 0.9186 |  |
| Sex – Male vs Female | 0.99 | 0.8964 | 1.05 | 0.88 | 1.25 | 0.6203 |  |
| Ethnicity – Hispanic vs Non Hispanic | 0.68 | 0.4973 |  |  |  |  |  |
| Age at Diagnosis | 1.02 | <.0001 | 1.03 | 1.02 | 1.04 | <.0001 | * |
| SEER Stage (Overall p-value) | -- | <.0001 | **--** | **--** | **--** | <.0001 | * |
| SEER Stage – Distant vs Localized | 2.12 | <.0001 | 2.22 | 1.77 | 2.77 | <.0001 | * |
| SEER Stage – Regional vs Localized | 1.44 | 0.0006 | 1.59 | 1.26 | 2.02 | 0.0001 | * |
| SEER Stage – Unstaged vs Localized | 2.19 | <.0001 | 1.86 | 1.35 | 2.56 | 0.0001 | * |
| Marital Status (Overall p-value) | -- | 0.0192 | **--** | **--** | **--** | 0.0912 |  |
| Marital – Divorced vs Married | 1.27 | 0.0637 | 1.32 | 1.01 | 1.73 | 0.0439 | * |
| Marital – Separated vs Married | 0.49 | 0.2192 | 0.43 | 0.13 | 1.37 | 0.1527 |  |
| Marital – Never Married vs Married | 1.30 | 0.0599 | 1.34 | 1.008 | 1.79 | 0.0499 | * |
| Marital – Unknown vs Married | 1.39 | 0.0207 | 1.24 | 0.85 | 1.79 | 0.2602 |  |
| Marital – Widowed vs Married | 1.31 | 0.0179 | 1.08 | 0.83 | 1.39 | 0.5765 |  |
| Insurance Status (Overall p-value) | -- | 0.0322 | **--** | **--** | **--** | 0.0420 | * |
| Insurance – Medicaid vs Private | 1.13 | 0.5242 | 0.89 | 0.58 | 1.36 | 0.5823 |  |
| Insurance – Medicare vs Private | 1.35 | 0.0016 | 0.78 | 0.60 | 1.01 | 0.0571 |  |
| Insurance – Other Insurance vs Private | 1.23 | 0.1964 | 1.17 | 0.83 | 1.64 | 0.3756 |  |
| Insurance – Uninsured vs Private | 1.39 | 0.2108 | 1.50 | 0.87 | 2.60 | 0.1451 |  |
| Area Type (Overall p-value) | -- | 0.3681 |  |  |  |  |  |
| Urban vs. Rural – Not Urban Adjacent | 0.79 | 0.1991 |  |  |  |  |  |
| Rural – Urban Adjacent vs. Rural | 0.85 | 0.4171 |  |  |  |  |  |
| County SDI Quartile (Overall p-value) | -- | 0.3399 |  |  |  |  |  |
| County SDI Quartile – Q2 vs Q1 | 0.95 | 0.6676 |  |  |  |  |  |
| County SDI Quartile – Q3 vs Q1 | 1.11 | 0.2624 |  |  |  |  |  |
| County SDI Quartile – Q4 vs Q1 | 1.14 | 0.2881 |  |  |  |  |  |
| *Neighborhood SDI Quartile (Overall p-value) | -- | 0.1736 | **--** | **--** | **--** | 0.5065 |  |
| Neighborhood SDI Quartile – Q2 vs Q1 | 1.02 | 0.8417 | 1.11 | 0.88 | 1.40 | 0.3916 |  |
| Neighborhood SDI Quartile – Q3 vs Q1 | 1.22 | 0.0637 | 1.17 | 0.92 | 1.47 | 0.1956 |  |
| Neighborhood SDI Quartile – Q4 vs Q1 | 1.17 | 0.1559 | 1.19 | 0.93 | 1.52 | 0.1763 |  |

## Supplemental Table 1. Cox proportional hazard models for time from diagnosis to death in patients with hepatocellular carcinoma including FPL 100 Score by quartile

| Effect | Univariable  Hazard Ratio | p-value | Multivariable  Hazard Ratio | 95% Hazard Ratio Confidence Limits | | p-value | (p<.05) |
| --- | --- | --- | --- | --- | --- | --- | --- |
| Race (Overall p-value) | -- | 0.0048 | -- | -- | -- | 0.0514 |  |
| Race – Black vs White | 0.91 | 0.0691 | 0.87 | 0.77 | 0.997 | 0.0455 | * |
| Race – Other vs White | 0.67 | 0.0049 | 0.78 | 0.56 | 1.09 | 0.1421 |  |
| Sex – Male vs Female | 1.14 | 0.0026 | 1.19 | 1.08 | 1.32 | 0.0007 | * |
| Ethnicity – Hispanic vs Non Hispanic | 1.43 | 0.0441 | 1.35 | 0.90 | 2.03 | 0.1459 |  |
| Age at Diagnosis | 1.01 | <.0001 | 1.02 | 1.01 | 1.02 | <.0001 | * |
| SEER Summary Stage (Overall p-value) | -- | <.0001 | -- | -- | -- | <.0001 | * |
| SEER Stage – Distant vs Localized | 3.23 | <.0001 | 3.35 | 2.97 | 3.78 | <.0001 | * |
| SEER Stage – Regional vs Localized | 2.02 | <.0001 | 2.03 | 1.83 | 2.24 | <.0001 | * |
| SEER Stage – Unstaged vs Localized | 2.32 | <.0001 | 2.09 | 1.75 | 2.50 | <.0001 | * |
| Marital Status (Overall p-value) | -- | <.0001 | -- | -- | -- | 0.0003 | * |
| Marital – Divorced vs Married | 1.05 | 0.4557 | 1.14 | 0.99 | 1.31 | 0.0766 |  |
| Marital – Separated vs Married | 1.13 | 0.4794 | 1.28 | 0.89 | 1.84 | 0.1813 |  |
| Marital – Never Married vs Married | 1.20 | 0.0019 | 1.31 | 1.15 | 1.48 | <.0001 | * |
| Marital – Unknown vs Married | 1.58 | <.0001 | 1.23 | 1.05 | 1.45 | 0.0088 | * |
| Marital – Widowed vs Married | 1.27 | 0.0006 | 1.23 | 1.05 | 1.43 | 0.0083 | * |
| Insurance Status (Overall p-value) | -- | <.0001 | -- | -- | -- | 0.0006 | * |
| Insurance – Medicaid vs Private | 1.20 | 0.0091 | 1.08 | 0.93 | 1.26 | 0.3304 |  |
| Insurance – Medicare vs Private | 1.42 | <.0001 | 1.14 | 1.00 | 1.29 | 0.0534 |  |
| Insurance – Other Insurance vs Private | 1.41 | <.0001 | 1.16 | 0.96 | 1.39 | 0.1205 |  |
| Insurance – Uninsured vs Private | 2.36 | <.0001 | 1.66 | 1.32 | 2.10 | <.0001 | * |
| Area Type (Overall p-value) | -- | 0.1490 |  |  |  |  |  |
| Urban vs. Rural – Not Urban Adjacent | 1.12 | 0.2583 |  |  |  |  |  |
| Rural – Urban Adjacent vs. Rural – Not Urban Adjacent | 1.21 | 0.0800 |  |  |  |  |  |
| County Federal Poverty Level | 1.00 | 0.6281 |  |  |  |  |  |
| *Neighborhood FPL Quartile (Overall p-value) | -- | 0.0185 | -- | -- | -- | 0.0184 | * |
| Neighborhood FPL Quartile – Q2 vs Q1 | 0.999 | 0.9838 | 0.93 | 0.83 | 1.05 | 0.2531 |  |
| Neighborhood FPL Quartile – Q3 vs Q1 | 1.09 | 0.1150 | 0.99 | 0.88 | 1.12 | 0.8795 |  |
| Neighborhood FPL Quartile – Q4 vs Q1 | 1.15 | 0.0091 | 1.14 | 1.01 | 1.30 | 0.0415 | * |
|  | | | | | | | |

## Supplemental Table 2. Cox proportional hazard models for time from diagnosis to death in patients with cholangiocarcinoma

* Model including race, sex, age, SEER summary stage, marital status, insurance status, and Neighborhood FPL 100 Score by Quartile

| Effect | Univariable Hazard Ratio | P-value | Multivariable Hazard Ratio | 95% Hazard Ratio Confidence Limits | | p-value | (p<.05) |
| --- | --- | --- | --- | --- | --- | --- | --- |
| Race – Black vs White | 1.10 | 0.5533 | 1.03 | 0.72 | 1.49 | 0.8627 |  |
| Sex – Male vs Female | 0.99 | 0.8964 | 1.04 | 0.87 | 1.24 | 0.6837 |  |
| Ethnicity – Hispanic vs Non Hispanic | 0.68 | 0.4973 |  |  |  |  |  |
| Age at Diagnosis | 1.02 | <.0001 | 1.03 | 1.02 | 1.04 | <.0001 | * |
| SEER Stage (Overall p-value) | -- | <.0001 | -- | -- | -- | <.0001 | * |
| SEER Stage – Distant vs Localized | 2.12 | <.0001 | 2.21 | 1.77 | 2.77 | <.0001 | * |
| SEER Stage – Regional vs Localized | 1.44 | 0.0006 | 1.59 | 1.26 | 2.01 | 0.0001 | * |
| SEER Stage – Unstaged vs Localized | 2.19 | <.0001 | 1.85 | 1.34 | 2.55 | 0.0002 | * |
| Marital Status (Overall p-value) | -- | 0.0192 | -- | -- | -- | 0.1107 |  |
| Marital – Divorced vs Married | 1.27 | 0.0637 | 1.31 | 1.00 | 1.72 | 0.0544 |  |
| Marital – Separated vs Married | 0.49 | 0.2192 | 0.46 | 0.14 | 1.47 | 0.1877 |  |
| Marital – Never Married vs Married | 1.30 | 0.0599 | 1.35 | 1.01 | 1.81 | 0.0413 | * |
| Marital – Unknown vs Married | 1.39 | 0.0207 | 1.22 | 0.84 | 1.77 | 0.2889 |  |
| Marital – Widowed vs Married | 1.31 | 0.0179 | 1.10 | 0.85 | 1.42 | 0.4665 |  |
| Insurance Status (Overall p-value) | -- | 0.0322 | -- | -- | -- | 0.0354 | * |
| Insurance – Medicaid vs Private | 1.13 | 0.5242 | 0.92 | 0.59 | 1.41 | 0.6937 |  |
| Insurance – Medicare vs Private | 1.35 | 0.0016 | 0.78 | 0.60 | 1.01 | 0.0590 |  |
| Insurance – Other Insurance vs Private | 1.23 | 0.1964 | 1.18 | 0.84 | 1.67 | 0.3375 |  |
| Insurance – Uninsured vs Private | 1.39 | 0.2108 | 1.52 | 0.88 | 2.63 | 0.1318 |  |
| County Federal Poverty Level | 1.00 | 0.2519 |  |  |  |  |  |
| Area Type (Overall p-value) | -- | 0.3681 |  |  |  |  |  |
| Urban vs. Rural – Not Urban Adjacent | 0.79 | 0.1991 |  |  |  |  |  |
| Rural – Urban Adjacent vs. Rural | 0.85 | 0.4171 |  |  |  |  |  |
| *Neighborhood FPL Quartile (Overall p-value) | -- | 0.4223 | -- | -- | -- | 0.7406 |  |
| Neighborhood FPL Quartile – Q2 vs Q1 | 1.11 | 0.3108 | 1.10 | 0.87 | 1.40 | 0.4253 |  |
| Neighborhood FPL Quartile – Q3 vs Q1 | 1.08 | 0.4968 | 1.01 | 0.80 | 1.28 | 0.9138 |  |
| Neighborhood FPL Quartile – Q4 vs Q1 | 1.19 | 0.1010 | 1.11 | 0.87 | 1.42 | 0.3828 |  |
|  | | | | | | |  |

**Supplemental Figure 1**. Conceptual Framework for the Relationship Between Area and Individual Social Determinants of Health and Cancer Mortality.

The social determinants of health are the upstream determinants of cancer mortality.

**Figure 1.** Social Deprivation Index Quartile 4 Deaths in patients with hepatocellular carcinoma.

The number of deaths in quartile 4 social deprivation index neighborhoods per100,000 thousand residents.

**Figure 2.** The Impact of the Cumulative Burden of Social Determinant Health on HCC Mortality.

**Panel A.** The SDOH that were associated with time to death. **Panel B.** Proportion alive at the end of the study period in the categories identified in panel A. **Panel C.** Survival curves for selected categories. **Panel D.** Log rank testing between selected categories.
